# Lnc-GAN1 expression is associated with good survival and suppresses tumor progression by sponging mir-26a-5p to activate PTEN signaling in non-small cell lung cancer

**DOI:** 10.1186/s13046-020-01819-0

**Published:** 2021-01-06

**Authors:** Rui-Qi Wang, Xiao-Ran Long, Ning-Ning Zhou, Dong-Ni Chen, Mei-Yin Zhang, Zhe-Sheng Wen, Lan-Jun Zhang, Fa-Zhong He, Zhi-Lin Zhou, Shi-Juan Mai, Hui-Yun Wang

**Affiliations:** 1 State Key Laboratory of Oncology in South China, Collaborative Innovation Center for Cancer Medicine, Sun Yat-Sen University Cancer Center, Guangzhou, 510060 China; 2 Zhuhai People’s Hospital (Zhuhai Hospital Affiliated with Jinan University), Zhuhai, 519000 China; 3 Department of Gynecology and Obstetrics, Renji Hospital, Medical School of Shanghai Jiaotong University, Shanghai, China; 4 Department of Medical Oncology, Sun Yat-Sen University Cancer Center, Guangzhou, 510060 China; 5 Department of Thoracic Oncology, Sun Yat-Sen University Cancer Center, Guangzhou, 510060 China

**Keywords:** Lung cancer, Lnc-GAN1, miR-26a-5p, PTEN

## Abstract

**Background:**

Long non-coding RNAs (lncRNAs) play vital roles in the development and progression of non-small-cell lung cancer (NSCLC); however, the role of most lncRNAs in NSCLC remains unknown. This study explored the clinical significance, biological function and underlying mechanism of lnc-GAN1 in NSCLC.

**Methods:**

With a custom lncRNA microarray we found that lnc-GAN1 is markedly downregulated in NSCLC tissues. Then lnc-GAN1 expression level was measured using qRT-PCR in NSCLC tissues and cell lines. Survival was assessed using the Kaplan-Meier method. The biological functions of lnc-GAN1 in lung cancer cells were evaluated in vitro and in vivo. RNA fluorescence in situ hybridization and subcellular localization assays revealed the subcellular distribution of lnc-GAN1 in cells. Bioinformatic analysis was adopted to predict miRNAs and signaling pathways regulated by lnc-GAN1. RNA immunoprecipitation and Dual-luciferase reporter assays were used to assess the interaction between lnc-GAN1 and miR-26a-5p in lung cancer cells.

**Results:**

lnc-GAN1 is downregulated in HCC tissues and associated with larger tumor size and poor overall survival and disease-free survival; its ectopic expression suppresses cell proliferation, colony formation, and cell cycle progression and induces apoptosis in NSCLC cells; it also inhibits tumor growth in the NSCLC xenograft model. We further proved that lnc-GAN1 is localized in cytoplasm and transcribed independently from its parental gene GAN. Mechanistically, lnc-GAN1 acts as a sponge for miR-26a-5p by two seed sequences, and the two non-coding RNAs have a negative relationship in NSCLC tissues; we further prove that PTEN is a direct target of miR-26a-5p and lnc-GAN1 inhibits cell cycle signaling pathway by activating PTEN, whose expression level correlated negatively with miR-26a-5p level but positively with lnc-GAN1 level in NSCLC samples.

**Conclusions:**

Lnc-GAN1 is downregulated and associated with poor survival of NSCLC patients, and mechanistically acts as a tumor suppressor via sponging and inhibiting miR-26a-5p to upregulate PTEN. This study provides a potential prognostic biomarker and treatment target for NSCLC.

## Background

Lung cancer is one of the most common malignancies and is a leading cause of cancer-related deaths worldwide, accounting for 2.1 million new cases and 1.8 million deaths per year [1]. The incidence and mortality of lung cancer in China has increased in recent decades, with 787,300 newly diagnosed cases and 631,000 deaths in 2019 [2]. Non-small cell lung cancer (NSCLC) is the predominant type of lung cancer including lung adenocarcinoma and lung squamous cell carcinoma, accounting for approximately 85% of all lung cancer cases [3]. Despite recent improvements in diagnosis and treatment, the prognosis for NSCLC remains poor with a five-year overall survival (OS) rate of 19.8% [1, 4, 5]. This poor prognosis is mainly due to three reasons: 1) most patients are diagnosed at an advanced stage; 2) high rates of recurrence and distant metastasis following surgical treatment; 3) absence of any effective and practical biomarkers for prognosis in clinical practice. Furthermore, the exact molecular mechanism underlying NSCLC development, progression and metastasis remains not fully clear. Therefore, a better understanding of the underlying molecular network and the identification of potential prognostic biomarkers and therapeutic targets are crucial to prolonging survival of patients with NSCLC.

Long non-coding RNAs (lncRNAs) are a group of transcripts > 200 bp with no protein-coding capacity [6]. These RNAs are involved in chromatin remodeling, as well as in transcriptional and post-transcriptional regulation during diverse biological processes, including cell cycle, cell differentiation, and immune responses [7, 8]; their molecular functions are generally associated with their subcellular localization patterns. Cytoplasmic lncRNAs are involved in translational regulation, whereby they modulate protein levels, whereas nuclear lncRNAs mainly regulate signal transductions through transcriptional regulation of the target genes [9, 10].

In recent years, the role of lncRNAs in cancer has received increasing attention. A growing body of evidence indicates that lncRNAs are associated with the development, progression, invasion, or metastasis of various tumors, including lung cancer, and play critical oncogenic or tumor-suppressive roles [11]. For instance, metastasis-associated lung adenocarcinoma transcript 1 (MALAT1) is one of the first lncRNAs shown to be associated with lung cancer, in which it plays a critical role during metastasis [12]. Notably, siRNA-mediated MALAT1 downregulation has been found to delay tumor growth and decrease metastasis in lung cancer xenograft models, indicating its potential as a therapeutic target [12]. In addition, another lncRNA, NEAT1, is upregulated in NSCLC and enhances the metastasis via the Wnt/β-catenin pathway [13], while high levels of “HOX transcript antisense RNA” (HOTAIR) have been associated with poor prognosis through increasing cell invasiveness by down-regulating HOXA5 expression [14]. Conversely, loss of GAS5-AS1 has been found to contribute to metastasis in lung cancer, and pan-histone deacetylase (HDAC) inhibitors inhibit NSCLC cell metastasis by increasing GAS5-AS1 expression [15]. AFAP1-AS1 is also associated with poor prognosis of lung cancer and promotes cell proliferation by inhibiting P21 [16], whereas LINC00673 promotes the proliferation, metastasis, and invasion of NSCLC by competitively binding to and suppressing miR-150-5p [17]. LincRNA-P21 has been shown to affect the prognosis of patients with NSCLC by promoting angiogenesis [18]. However, despite these reports, lncRNA functions and expression patterns in patients with NSCLC are still poorly understood.

Previously, we have performed lncRNA microarray analysis on the tumors and adjacent healthy tissues from 194 patients with NSCLC to characterize the lncRNA expression profile in lung cancer [19]. Consequently, lnc-GAN1 was found to be significantly downregulated in NSCLC tissues and was associated with the poor survival of patients. Accordingly, it may serve as a potential prognostic factor for patients with NSCLC.

Lnc-GAN1 is derived from the 3′-untranslated region (3’UTR) of GAN gene. This gene encodes gigaxonin, a cytoskeletal component involved in filament processing in neural cells and fibroblasts. Gigaxonin functions as an E3 ubiquitin ligase adaptor protein and mediates the ubiquitination and subsequent proteasomal degradation of the targeted proteins [20–22]. Although several 3′UTR-associated lncRNAs have been identified, their functions remain poorly understood [23, 24]. In this study, we found that lnc-GAN1 suppressed proliferation and cell cycle progression and induced apoptosis in lung cancer cells. Moreover, lnc-GAN1 was shown to act as an endogenous miR-26a-5p sponge, thereby upregulating the protein level of the miR-26a-5p target gene PTEN that mediates tumor-suppression in lung cancer.

## Methods

### Clinical samples and cell lines

Human NSCLC specimens were obtained from 194 patients who underwent radical lung cancer resection at the Sun Yat-Sen University Cancer Center between 2003 and 2008. Corresponding adjacent noncancerous lung tissues were simultaneously obtained from these patients. All the samples were independently diagnosed by two pathologists and stored at -80 °C. The immortalized human lung cell line Beas-2B and the human NSCLC cell lines A549, H460, H1299, H1650, 95D, and H1975 were maintained in the State Key Laboratory of Oncology in South China and authenticated using Short Tandem Repeat profiling. All the cell lines were tested for Mycoplasma contamination (all negative). The cells were cultured in RPMI-1640 medium (GIBCO, Grand Island, NY, USA) with 10% fetal bovine serum at 37 °C with 5% CO_2_.

### RNA extraction and qRT-PCR

Total RNA was extracted from lung cancer and adjacent lung tissues and cell lines using TRIzol reagent according to the manufacturer’s instructions (Invitrogen, Carlsbad, CA, USA). RNA quantity and quality were assessed using a NanoDrop™ spectrophotometer (Thermo Fisher Scientific, Waltham, MA, USA), and 1 μg of total RNA was reverse-transcribed into first-strand cDNA using a cDNA Reverse Transcription Kit (Promega, Madison, WT, USA). RNA levels were measured by quantitative PCR using Power SYBR Green PCR Master Mix (Promega) and normalized to GAPDH mRNA level for lncRNAs or protein-coding genes and to U6 snRNA level for microRNAs. The primers used for qRT-PCR are listed in Additional file 1: Table S1.

### Plasmids and transfection

The lentiviral vector pGLV5 containing full-length lnc-GAN1 was purchased from GenePharma (Suzhou, China) and transfected into A549 and H460 cells with 8 μg/mL of Polybrene (Sigma, St Louis, Missouri, USA). Stable cell lines were selected using 4 μg/mL of puromycin. H1650 and H1299 cells were transiently transfected with a scramble siRNA or siRNAs targeting lnc-GAN1 (siRNA-1 or siRNA-2) by using Lipofectamine 3000 reagent (Invitrogen, Carlsbad, CA, USA) in Opti-MEM® I Reduced-Serum Medium (OPTI-MEM) (Gibco, Thermo Fisher Scientific, USA), and the siRNA sequences are shown in Additional file 1: Table S1. The efficiency of lnc-GAN1 overexpression or knockdown was verified by qRT-PCR. In the knockdown experiment, lnc-GAN1-siRNA-2 had better inhibitory effect among the siRNAs that were tested. Based on this siRNA, a shRNA was synthesized and inserted into pLKO vectors to generate stable lnc-GAN1-knockdown cells.

### Cell viability and colony formation assays

Cell proliferation assays were performed using a Cell Counting Kit-8 (CCK8) (APExBio, Houston, TX, USA) according to the manufacturer’s instructions. Cells were plated in 96-well plates at a density of 1000 cells/well and cultured at 37 °C with 5% CO_2_ for 4 days. The optical density of each well was measured at 450 nm using a Synergy HT Multi-Mode Microplate Reader (BioTek, Winooski, VT, USA). For the colony formation assay, cells were plated in six-well plates (2000 cells/well) and cultured at 37 °C with 5% CO_2_ for 2 weeks. Colonies were fixed with methanol and stained with 0.1% crystal violet for 30 min. All experiments were performed in triplicate for each cell line.

### Flow cytometry analysis of cell cycle and apoptosis

To analyze the cell cycle, cultured cells in a six-well plate were harvested after 24 h of serum starvation and fixed overnight in 75% ethanol at 4 °C. The cells were then washed in phosphate-buffered saline (PBS) three times, treated with RNase A, and incubated with Propidium Iodide (PI) staining solution for 30 min. Flow cytometry was performed using an ACEA NovoCyte Flow Cytometer (Agilent Technologies, Santa Clara, CA, USA). To determine the rate of apoptosis, cultured cells were harvested and stained with Annexin V-FITC and PI according to the manufacturer’s instructions (eBioscience, Shanghai, China). The stained cells were then analyzed by flow cytometry to distinguish between viable, early apoptotic, and late apoptotic cells (dead cells). The percentage of apoptotic cells was recorded for analysis. The above experiments were done on each cell line in triplicate.

### Tumor xenograft model

Athymic BALB/c nude mice (6-week-old) were housed in laminar air flow cabinets under pathogen-free conditions. A549 or H460 cells (1 × 10^6^) with stably overexpressing lnc-GAN1 or control vector were injected into the flank of a mouse (*n* = 6 per group). After 24 days, the mice were sacrificed under anesthesia, and the tumor tissues were harvested and weighted. The maximum diameter and width of tumors were measured with a caliper. Tumor volume was calculated with the following formula: Volume = (length × width^2^)/2. Lnc-GAN1 levels in the tumors were determined by qRT-PCR [25]. The animal experiments were approved by the Animal Care and Use Committee of Sun Yan-Sen University Cancer Center.

### Immunohistochemical (IHC) analysis

The xenograft tumor samples were embedded in paraffin, cut into 4-μm sections in a microtome, and incubated with an antibody against Ki67 (Proteintech, Wuhan, China) with DAB staining according to the manufacturer. The percentage of apoptotic cells was recorded using an Olympus DP73 digital microscope camera (Olympus, Tokyo, Japan).

### RNA fluorescence in situ hybridization (FISH)

NSCLC cells were seeded in a 35-mm Petri dish and cultured overnight at 37 °C with 5% CO_2_. The NSCLC tissues were sliced into 4-μm thick sections. A549 and H1650 cells were washed with PBS and fixed in 4% formaldehyde for 15 min. Then cells were treated with Triton X-100 and dehydrated with a series of ethanol concentrations. Cy3-labeled lnc-GAN1 probe was designed by and purchased from RiboBio Company (Guangzhou, China). RNA FISH was conducted three times by using the RiboTM Fluorescent In Situ Hybridization Kit (RiboBio RN: R11060.7) following the manufacturer’s instructions [26]. Next, cells were washed with PBS three times and photographed using a Zeiss LSM880 fluorescence microscope (Carl Zeiss Microscopy GmbH, Jena, Germany).

### Subcellular fractionation of lnc-GAN1

Cytoplasmic and nuclear fractionation and RNA isolation were performed by using a PARIS^TM^ Kit (AM1921, Ambion, Austin, TX, USA). The H1299 or H1650 cells were seeded in a 10-cm Petri dish and collected according to the manufacturer’s instructions. The subcellular distribution of Lnc-GAN1 was assessed by qRT-PCR and GAPDH and U1 were used as cytoplasmic and nuclear controls, respectively. lnc-MALAT1 was used as a positive control since it has been reported to be localized in the nucleus [27].

### Transcriptional profiling and gene function and pathway analysis

Total RNA was extracted from lnc-GAN1-overexpressing and negative control A549 cells with TRIzol Reagent. RNA quantity and integrity were evaluated using an Agilent Bioanalyzer 2100 (Agilent Technologies, Palo Alto, USA). Transcriptional profiling was performed using an Agilent SurePrint G3 Human Gene Expression Microarray, which was conducted by Shanghai Biotechnology Company (Shanghai, China). After quality control, raw data were analyzed using the limma package in R language. Differentially expressed genes were identified using the DESeq2 Bioconductor package in R language (fold change ≥2, *P*-value < 0.05, FDR filtering < 0.05). Gene ontology and Kyoto encyclopedia of genes and genomes (KEGG) pathway analyses were conducted using the Cluster Profiler of DESeq2 Bioconductor package.

### Western blotting

Western blotting with Chemiluminescent detection for 30 μg protein extracts was performed as previously described by us [28, 29] with the following antibodies: anti-PTEN (1:1000 Cell Signaling Technology [CST], Danvers, MA, USA), anti-CDK4 (1:1000, CST), anti-cyclin D1 (1:1000, CST), and anti-GAPDH (1:10000, Wuhan, Proteintech).

### Dual-luciferase reporter assay

Full-length lnc-GAN1 and PTEN 3′UTR sequences containing complementary sequence for miR-26a-5p seed region were amplified from a human cDNA library derived from NSCLC tissues and cloned into the 3′ end of the pGL3c-basic luciferase vector (Genechem, Shanghai, China). Lnc-GAN1 with mutated complementary sequence for miR-26a-5p seed sequence was generated by PCR. H1650 and A549 cells were seeded overnight in 24-well plates and co-transfected with 500 ng of wild or mutated pGL3c-lnc-GAN1 vector or pGL3c-control, 40 pmol of miR-26a-5p mimic, and 10 ng of the pRL-TK plasmid (internal control) per well using Lipofectamine 3000 (Invitrogen). After 48 h, luciferase activity was analyzed using a TransDetect® Dual Luciferase (Firefly) Reporter Assay Kit (Transgen, Beijing, China) according to the manufacturer’s instructions.

### RNA immunoprecipitation (RIP)

Cells were lysed using a RIP Kit (Bes5101, BersinBio, Guangzhou, China) to extract the RNAs that were bound by specific proteins. Briefly, 1 × 10^7^ cells were harvested and resuspended in PBS supplemented with 1% Protease Inhibitor Cocktail (Thermo Scientific™, Waltham, USA). Cell lysates were incubated in RIP buffer on ice by following the manufacture’s instruction, and anti-AGO2 antibody (CST, USA) was employed to capture the mixture of AGO2 protein and its bound RNAs. Co-immunoprecipitated RNAs were isolated using the TRIzol Reagent and analyzed by qRT-PCR with normalization to total input RNA levels.

### Co-immunoprecipitation (Co-IP)

H1650 cells were lysed in Pierce™ IP Lysis Buffer (Invitrogen, CA, USA) supplemented with Protease Inhibitor Cocktail. For immunoprecipitation, cell Lysates were incubated under shaking conditions with anti-PTEN, anti-CDK4, anti-Cyclin D1 or rabbit IgG antibodies (CST, MA, USA) overnight at 4 °C, and then Protein A/G agarose beads were added and incubated for 4 h. Beads were washed with lysis buffer three times. The immunoprecipitated complexes were then analyzed by western blot.

### Chromatin immunoprecipitation (ChIP)

ChIP assays were performed as described previously by us [28]. Briefly, H1650 cells were cross-linked with 1% formaldehyde for 10 min at 25 °C and then cells were lysed and subjected to ultrasonic crushing to obtain 200–800 bp DNA fragments. After incubation with anti-Pol II (2 mg, CST) or IgG antibodies and Protein A/G magnetic beads for 16 h at 4 °C, immunoprecipitation was performed. DNA fragments were purified using a DNeasy Blood & Tissue Kit (Qiagen, Hilden, Germany) and quantified by qPCR in triplicate (Roche, Basel, Switzerland). Primer sequences for the lnc-GAN1 promoter are shown in Additional file 1: Table S1.

### Statistical analysis

Experimental assays were conducted in triplicate, and the data are presented as mean (SD). Two-tailed Student’s t-test was used to assess the relationships among gene sets. The CCK8 assays were analyzed by means of two-way ANOVA. Patient survival was analyzed using the Kaplan-Meier method and assessed using the Log-Rank test. Statistical analysis was performed using the Statistical Program for Social Sciences 23.0 (SPSS, CA, USA) and the results were presented using GraphPad Prism 8.0 (GraphPad Software, San Diego, CA, USA). Results with *P*-values of < 0.05 were considered significant (* *P* < 0.05, ** *P* < 0.01, *** *P* < 0.001).

## Results

### Lnc-GAN1 is downregulated and correlates with poor prognosis in NSCLC

**Fig. 1:**
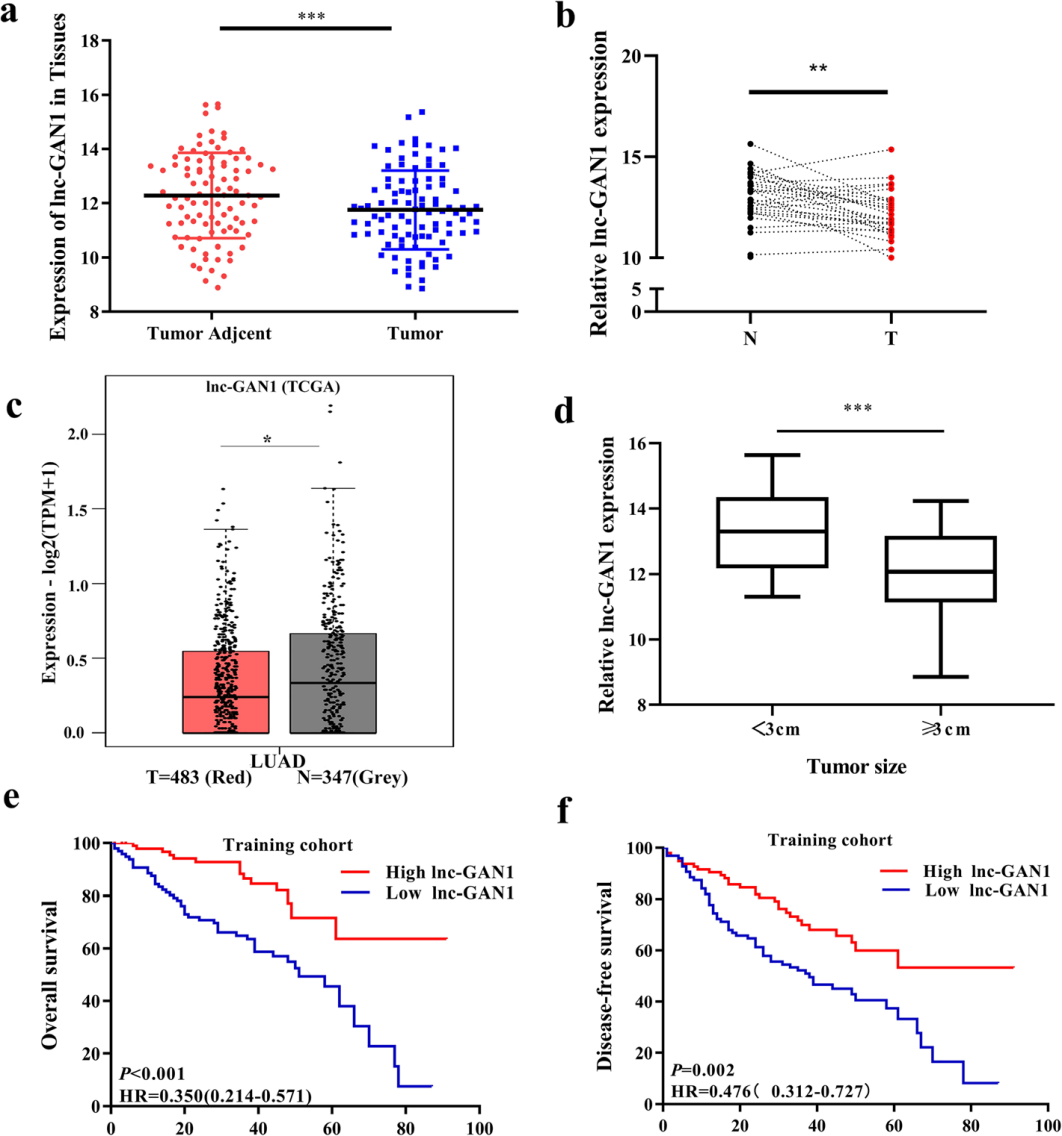
Lnc-GAN1 is downregulated in NSCLC tissues. **a** Lnc-GAN1 is significantly downregulated in 194 NSCLC tissues compared with 100 matched adjacent normal lung tissue detected by lncRNA microarray (*** *P* < 0.001). **b** lnc-GAN1 levels in 30 pairs NSCLC and adjacent tissues were measured by real-time qRT-PCR. lnc-GAN1 level is significantly lower in NSCLC tissues than in the paired adjacent normal lung tissues. (* *P* < 0.05). **c** lnc-GAN1 expression level is lower in lung adenocarcinomas (*n* = 483) than in the normal lung tissues (*n* = 347) obtained from TCGA (* *P* < 0.05). **d** lnc-GAN1 is highly expressed in NSCLC tumors less than 3 cm than in those larger than or equal to 3 cm (*** *P* < 0.001). **e**, **f** Kaplan-Meier survival analysis suggests that NSCLC patients with high lnc-GAN1 levels had better overall survival (OS) and disease-free survival (DFS) than those with low lnc-GAN1 expression

Previously, we performed lncRNA microarray analysis on 194 NSCLC samples and 100 paired normal lung tissues to determine lncRNA expression profiles, and identified 305 differentially expressed lncRNAs between NSCLC and normal lung tissues, of which 138 LncRNAs were upregulated and 167 downregulated in NSCLC tissues [19]. In the downregulated lncRNAs, lnc-GAN1 is one of the most significantly downregulated lncRNAs in NSCLC tissues compared with adjacent normal lung tissue (Fig. 1a). To verify the microarray result, we used qRT-PCR to detect lnc-GAN1 expression levels in 30 paired NSCLC and adjacent lung tissues. The result shows that lnc-GAN1 expression is significantly decreased in the 30 NSCLCs compared with that in the paired normal lung tissues (Fig. 1b), which is consistent with the microarray result, implying that microarray data is reliable and reproducible. To further confirm this result, we downloaded and analyzed the lnc-GAN1 expression data from TCGA database and found that lnc-GAN1 expression level was also reduced in lung adenocarcinoma compared with normal lung tissues (Fig. 1c), which is concordant with the result in our study. In the clinical analysis, we find that lnc-GAN1 levels are significantly lower in larger tumors (maximum diameter ≥ 3 cm) than in small tumors (< 3 cm) (Fig. 1d). To assess the significance of lnc-GAN1 expression in the survival of NSCLC patients, we first divided 194 patients into low- and high-lnc-GAN1 groups based on the median lnc-GAN1 level. Next, we performed Kaplan-Meier survival analysis to compare the association between lnc-GAN1 levels and patient outcomes. We find that the patients with low lnc-GAN1 expression have significantly poorer overall survival (OS) and disease-free survival (DFS) than those with high lnc-GAN1 expression (Fig. 1e, f). More important, Cox regression analysis revealed that lnc-GAN1 is a significant independent prognostic factor for overall survival (OS) in NSCLC patients (Table S2), indicating that lnc-GAN1 might be a potential survival predictor for NSCLC patients.

### Lnc-GAN1 inhibits oncogenic growth of NSCLC cells in vitro and in vivo

**Fig. 2:**
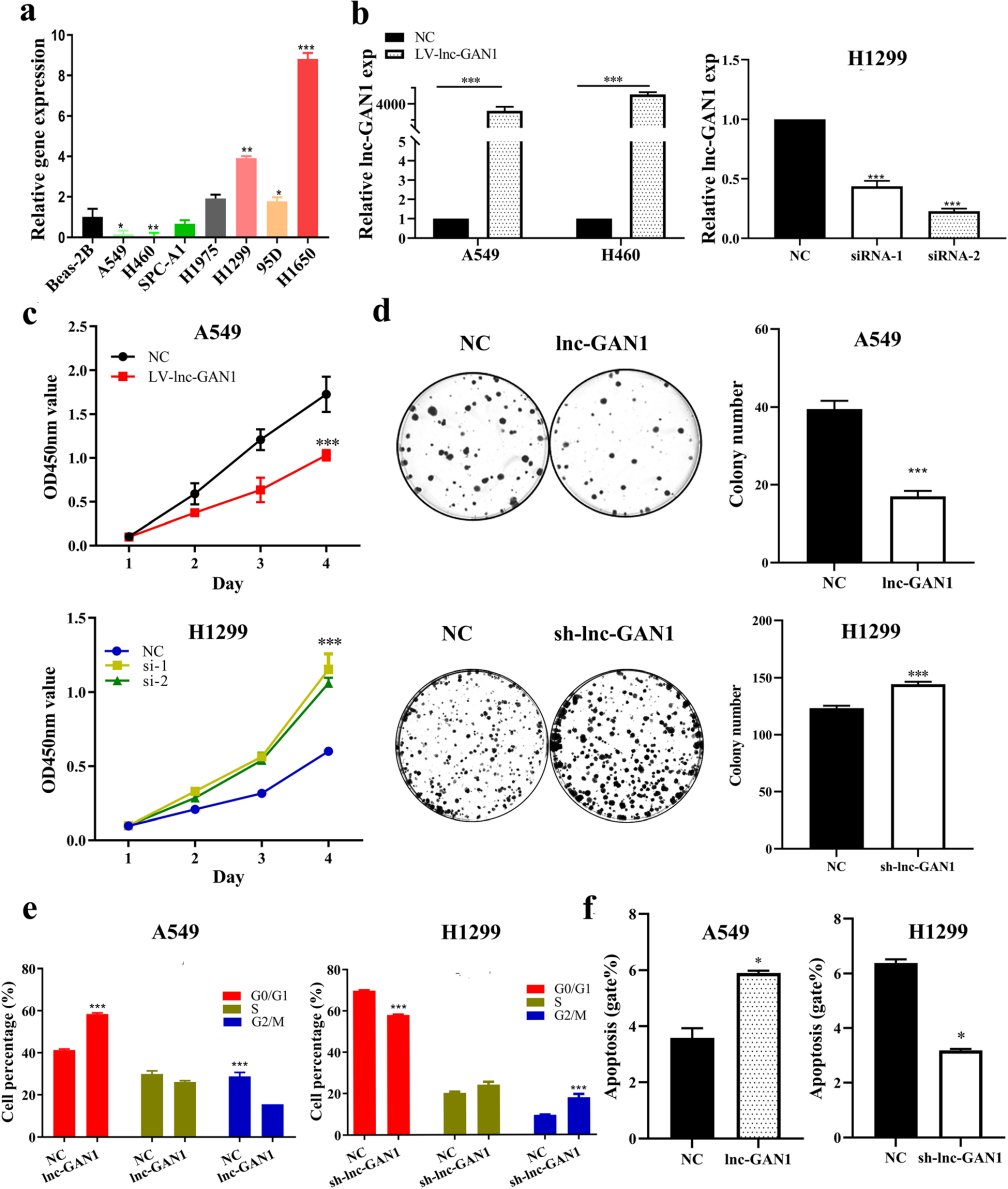
Lnc-GAN1 functions as a tumor suppressor for NSCLC cells in vitro. **a** Lnc-GAN1 levels were measured by real-time qRT-PCR in the NSCLC cell lines A549, H-460, H1299, H1650, SPC-A1, H1975, 95D, and in a human lung epithelial cell line BEAS-2B. **b** The histograms represent Lnc-GAN1 expression levels detected by qRT-PCR in A549 and H460 cells with overexpression of lnc-GAN1 or control vector, and in H1299 transfected with siRNA against lnc-GAN1 or control siRNA. **c** Proliferation curves of A549 cells with overexpression of lnc-GAN1 or control vector and H1299 cells transfected with sh-lnc-GAN1 or control shRNA, as determined by CCK8 assay. The results show that overexpressed lnc-GAN1 represses cell proliferation in A549 cells and sh-lnc-GAN1 has the reverse effects in H1299 cells. **d** Colony formation assay was performed to evaluate the oncogenic growth of A549 cells overexpressing lnc-GAN1 or control vector and H1299 cells transfected with sh-lnc-GAN1 or control. The right histograms denote the colony number of and of the A549 and H1299 cells, respectively, indicating that overexpressed lnc-GAN1 inhibits colony formation of A549 cells and sh-lnc-GAN1 enhances colony formation of H1299 cells. **e** The histograms designate percentage changes of G0/G1, S, and G2/M phases in A549 cells overexpressing lnc-GAN1 or control vector and H1299 cells transfected with sh-lnc-GAN1 or control, as detected by flow cytometry assay. The result demonstrates that overexpressed lnc-GAN1 induces cell cycle arrest in G0/G1 phase of A549 cells, and silencing lnc-GAN1 has the reverse effects on H1299. **f** The histograms represent the apoptosis percentage of A549 overexpressing lnc-GAN1 or control vector and H1299 cells transfected with sh-lnc-GAN1 or control shRNA, as detected by flow cytometry assay. The result suggests that overexpressed lnc-GAN1 induces apoptosis in A549 cells, and silencing lnc-GAN1 has the reverse effects on H1299 cells. Data represent mean ± SD of three independent experiments (*P < 0.05; ***P < 0.001, by Student’s *t*-test)

**Fig. 3:**
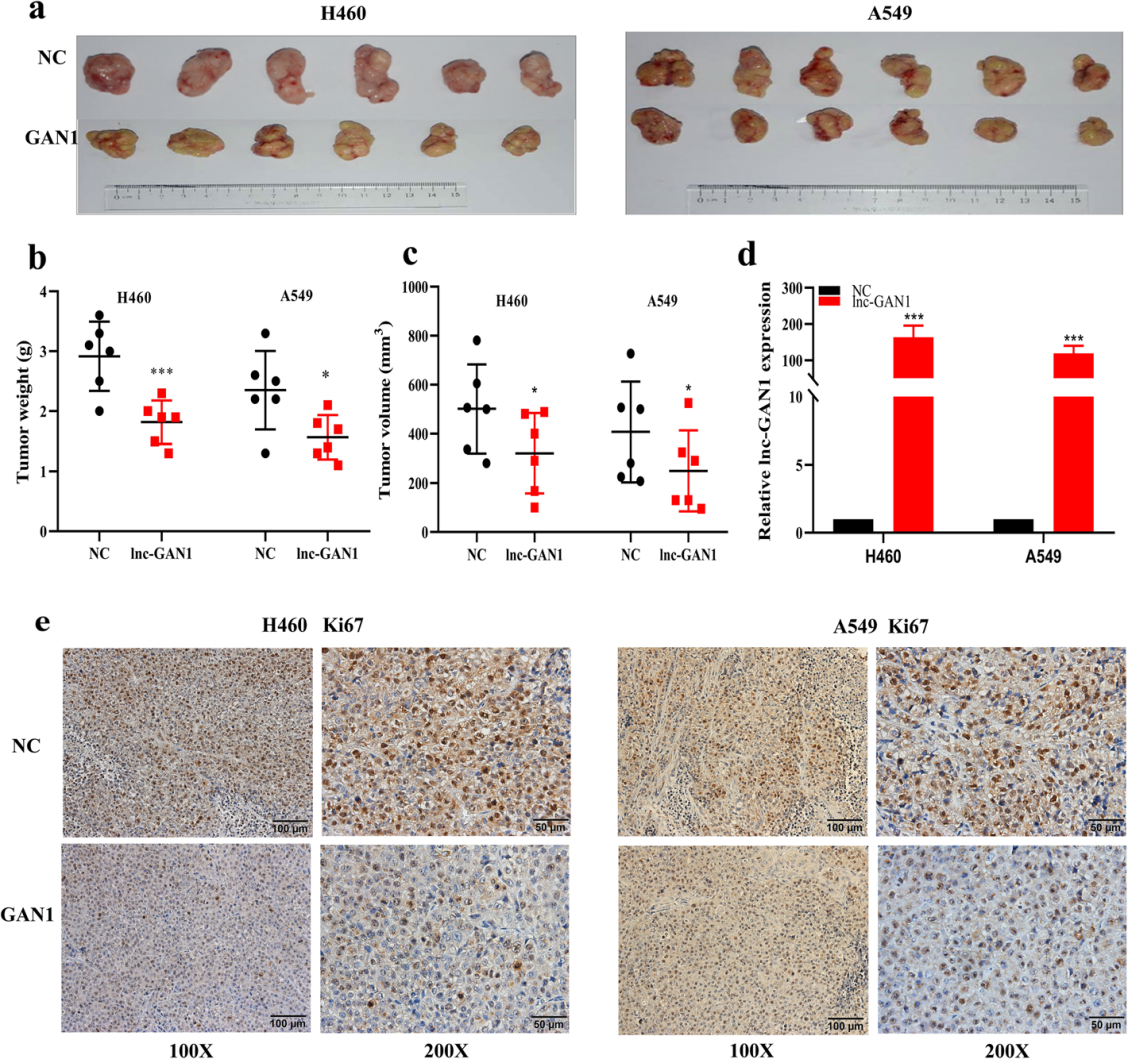
Lnc-GAN1 represses NSCLC cell growth in vivo. **a** The tumor masses are derived from H460 and A549 cells with overexpression of lnc-GAN1 or control vector, which were subcutaneously inoculated into the flank region of 6 nude mice, respectively. Upper penal: Negative controls; lower penal: lnc-GAN1 overexpression. **b**, **c** The weights (**b**) and volume (**c**) of the tumors derived from H460 and A549 cells with overexpression of Lnc-GAN1 or control vector, respectively. **d** Lnc-GAN1 extracted from  Xenotransplanted tumors was detected by qRT-PCR. **e** Ki-67 protein levels were detected with IHC in tumors derived from A549 or H460 cells with overexpressing lnc-GAN1 or control vector. *P < 0.05; ***P < 0.001

The above clinical analysis demonstrates that lnc-GAN1 is downregulated and plays a tumor suppressive role in NSCLC. To confirm the tumor suppressor role of lnc-GAN1 in vitro and in vivo, we first examined lnc-GAN1 levels in 7 NSCLC cell lines by qRT-PCR and found that 3 cell lines including A549, H460 and SPC-A1 cells had lower lnc-GAN1 level than the immortalized lung cell line Beas-2B, which is basically consistent with the result in NSCLC tissues, whereas another 4 cell lines including H1975, H1299, 95D and H1650 cells had higher levels (Fig. 2a). Therefore, H460 and A549 cells were selected to generate the stable cell lines with overexpression of lnc-GAN1, while H1299 and H1650 cells were employed to construct the cell lines with downregulation of lnc-GAN1. The expression level of Lnc-GAN1 in these stable cell lines was verified by qRT-PCR (Fig. 2b and Additional file 2: Fig. S1a). Next, we investigated the tumor suppressor role of lnc-GAN1 on these NSCLC cells with CCK8 and colony formation assays. The results reveal that cell proliferation and colony formation are significantly decreased when lnc-GAN1 is overexpressed in H460 and A549 cells, but is markedly increased when lnc-GAN1 is knocked down in H1299 and H1650 cells (Fig. 2c, d and Additional file 2: Fig. S1b-c), indicating that lnc-GAN1 can inhibit oncogenic growth of NSCLC cells and functions as a tumor suppressor, which is consistent with the result obtained in the above clinical analysis. To explore how lnc-GAN1 slows down the growth of lung cancer cells, we conducted cell cycle and apoptosis analyses on these NSCLC cells with flow cytometry. We observed the increased percentage of cells in G0/G1 phase and the decreased percentage in G2/M phase in H460 and A549 cells with lnc-GAN1 overexpression, and the opposite phenotypes in H1299 and H1650 cells with lnc-GAN1 downregulation (Fig. 2e and Additional file 2: Fig. S1d); meanwhile, overexpressed lnc-GAN1 significantly increased the apoptosis in H460 and A549 cells whereas downregulated lnc-GAN1 decreased the apoptosis in H1299 and H1650 cells (Fig. 2f and Additional file 2: Fig. S1e). These results suggest that lnc-GAN1 inhibits oncogenic growth of lung cancer cells in vitro by arresting cell cycle and inducing apoptosis. Finally, to determine if lnc-GAN1 has growth inhibitory effect in vivo*,* we generated a xenograft model by subcutaneously implanting H460 or A549 cells stably overexpressing lnc-GAN1 or carrying a control vector into BALB/c nude mice. After 24 days, the mice were euthanized, and tumor tissues were harvested (Fig. 3a). Consistent with the in vitro results, both the tumor weight and volume were significantly lower in the lnc-GAN1 overexpressing group than in the control group (Fig. 3b, c). Then we compared the lnc-GAN1 expression between xenograft tumors derived from NSCLC cells with or without lnc-GAN1 overexpression; the results are presented in Fig. 3d. In addition, IHC staining reveals significantly lower Ki-67 expression (growth index) in the tumors derived from the cells with high lnc-GAN1 expression than in tumors derived from the control cells (Fig. 3e), indicating that the tumor growth is inhibited by lnc-GAN1 overexpression. Altogether, these results indicate that lnc-GAN1 inhibits the growth of lung cancer cells in vitro and in vivo.

### Lnc-GAN1 is expressed independent of GAN transcription

**Fig. 4:**
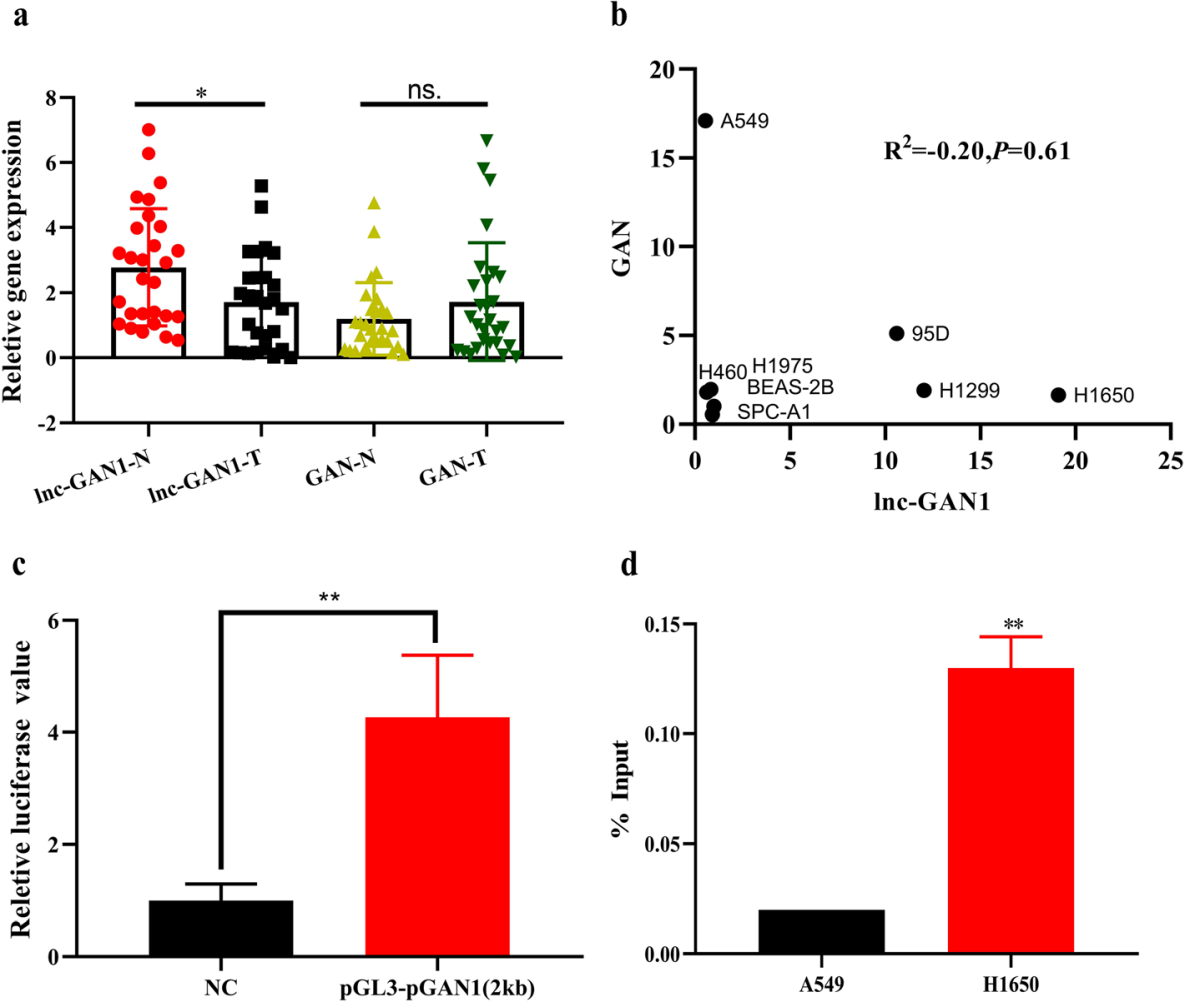
Lnc-GAN1 is expressed independent of its parent gene GAN. **a** Lnc-GAN1 expression is lower in 30 NSCLC tissues than in paired normal lung tissues, while its parent gene GAN expression is not different between the same NSCLC and paired normal lung tissues. **b** There is not a correlation between Lnc-GAN1 and GAN expressions in 8 cell lines. **c** In dual-luciferase reporter assay, A549 cells with the reporter containing 2000-bp DNA sequence upstream of lnc-GAN1 produce significantly higher Luciferase activity than A549 cells with control reporter, indicating that the 2000-bp sequence harbors promoter binding site. Luciferase activity was calculated as a firefly/renilla luciferase ratio and expressed as mean ± SD of three independent experiments. **d** Chromatin immunoprecipitation (ChIP) analysis on H460 and H1650 cells with Pol II antibody and the precipitated DNAs were amplified by PCR with the primers for the 2000-bp DNA sequence upstream of lnc-GAN1. H1650 cells with high expression of lnc-GAN1 show remarkably higher PCR product than H460 cells with very low expression of lnc-GAN1, suggesting that this DNA sequence has high Pol II activity in H1650 cells

Both clinical and experimental studies indicate that lnc-GAN1 plays a suppressor role in lung cancer. Next, we wanted to elucidate the underlying mechanism by which lnc-GAN1 inhibits growth of lung cancer cells. When searching the PubMed database, we could not find any report on lnc-GAN1. In the LNCipedia database, lnc-GAN1 is located on the long arm of chromosome 16 (16q23.2), and its sequence is overlapped with the DNA sequence of GAN 3’UTR (Additional file 2: Fig. S2a). To explore the relationship between lnc-GAN1 and GAN expressions, we measured GAN mRNA levels in 30 pairs of NSCLC and normal lung tissues by qRT-PCR. The results show no significant difference in the GAN mRNA levels between 30 pairs of NSCLC and normal lung tissues (Fig. 4a right), which is different from that of lnc-GAN1 expression in the 30 paired tumor and lung tissues (Fig. 4a left). Then we compared the expression levels of lnc-GNA1 and GAN mRNA in 8 cell lines and found that lnc-GAN1 expression level was completely unrelated to GAN mRNA level (Fig. 4b). These results suggest that lnc-GAN1 expression may be independent from GAN expression although lnc-GAN1 DNA sequence is located within the GAN gene. If lnc-GAN1 is independently transcribed from the GAN gene on the genome, it must have a promoter region upstream of the transcriptional start site that should be located in the 3’UTR sequence of the GAN gene. To verify this assumption, we performed Neural Network Promoter Prediction (NNPP, an online program) analysis on the 2000-bp DNA sequence upstream of lnc-GAN1 transcriptional start site to find possible promoter binding sites using a cut-off value of 0.90. The predicted result by NNPP program shows 5 promoter binding sites in 355–1500 bp upstream of the lnc-GAN1 (Additional file 2: Fig. S2b). To verify these possible promoter sites, we conducted Luciferase reporter assay on the 2000-bp DNA sequence upstream of lnc-GAN1 in A549 and H1650 cells. The result indicates that the relative fluorescence intensity of luciferase reporter containing the 2000-bp upstream sequence is significantly higher than that of the negative control reporter (Fig. 4c), indicating that the 2000-bp sequence contains promoter binding site(s). To start a transcription, the promoter binding region will recruit RNA polymerase II (Pol II) and transcription factors. Thus, we conducted ChIP assay with antibody against Pol II in H1650 cells, with the highest endogenous expression of lnc-GAN1, and A549 cells, with the lowest endogenous expression of lnc-GAN1, and the precipitated DNA fragments were amplified by quantitative PCR with primers for the 2000-bp sequence (Additional file 2: Fig. S2c). The results show a much higher amount of PCR product from H1650 cells with endogenous high lnc-GAN1 expression compared with that from A549 cells with endogenous low lnc-GAN1 expression (Fig. 4d), indicating that the DNA fragment contains Pol II binding sequence. Taken together, these results suggest that lnc-GAN1 can be independently transcribed from DNA locus of GAN gene.

### Lnc-GAN1 directly interacts with mir-26a-5p as a sponge in NSCLC

**Fig. 5:**
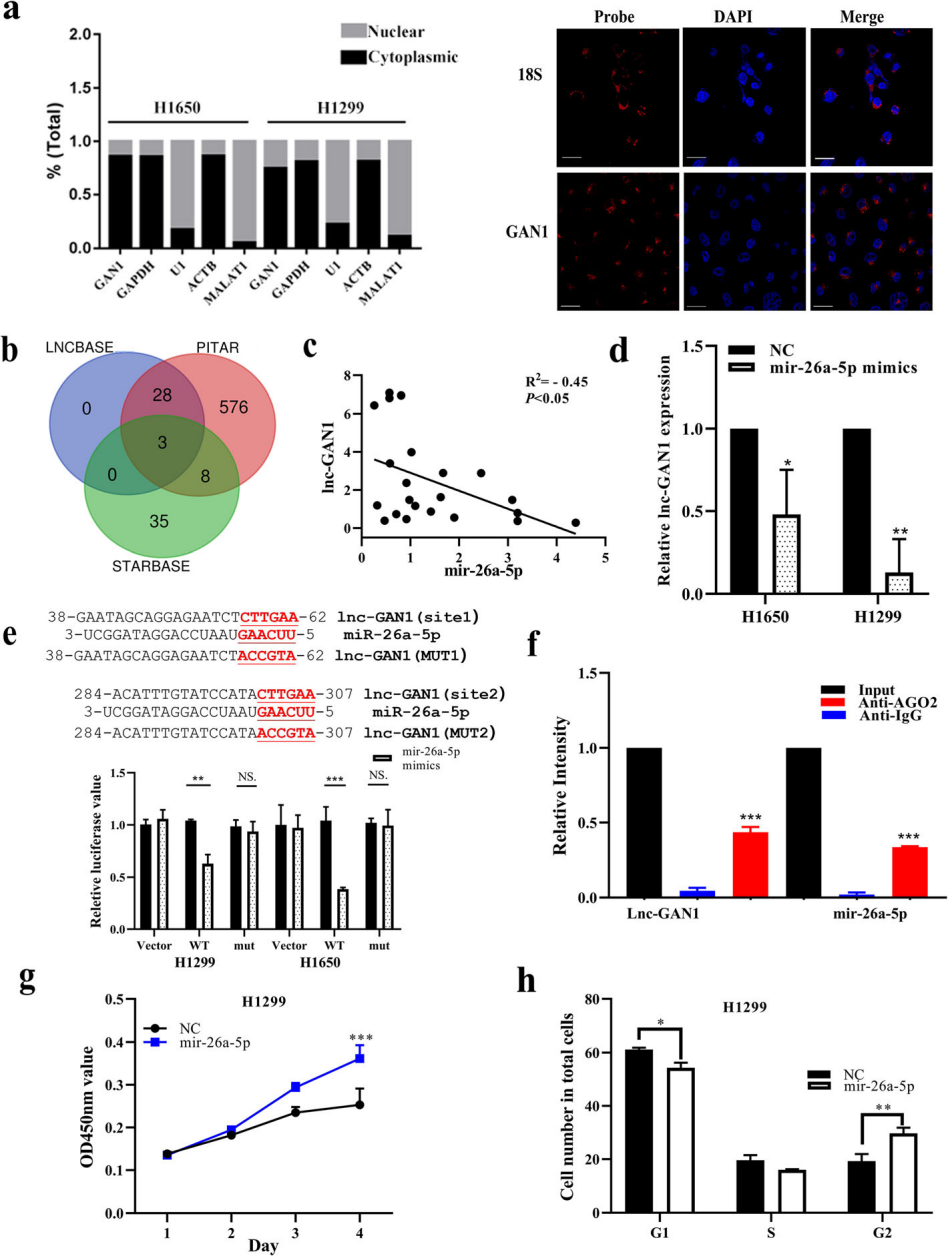
Lnc-GAN1 located in cytoplasm sponges and inhibits miR-26a-5p. **a** Left-Panel: Lnc-GAN1 is mainly located in the cytoplasm of H1299 and H1650 cells, as measured by real-time qPCR on cytoplasm and nucleus, respectively. GAPDH mRNA was used as a cytoplasmic control, while U1 snRNA was used as a nuclear control. Right-Panel: lnc-GAN1 was visually observed in the cytoplasm of H460 cells, as shown by FISH. 18S was used as the positive control in cytoplasm. **b** Venn diagram shows 3 shared predicted targets (miRNAs) of lnc-GAN1 by three databases, including miR-26a-5p, mir-26b-5p and mir-1297. **c** There is a significantly negative correlation between lnc-GAN1 and mir-26a-5p levels in 30 NSCLC samples, as determined by qRT-PCR (R^2^= − 0.45, *P* < 0.05). **d** Ectopic miR-26a-5p expression significantly reduced lnc-GAN1 mRNA levels in H1299 and H1650 cells (* *P* < 0.05), as measured by qRT-PCR. **e** Upper-Panel: The predicted wild-type seed sequences of lnc-GAN1 complementary with miR-26a-5p and investigator-designed mutated seed sequences. Lower-panel: Histogram shows that when miR-26a-5p was overexpressed in H1299 or H1650 cells, the luciferase activity of cells with wild-type seed sequence of lnc-GAN1 was sharply reduced compared with that of control cells, but not changed in cells with mutant miR-26a-5p binding sites, as assayed by Dual-luciferase reporter assays, indicating that there are direct binding sites between lnc-GAN1 and miR-26a-5p. **f** RIP was performed on H1650 cell lysates with mouse anti-AGO2 or IgG. The precipitated lnc-GAN1 and miR-26a-5p were evaluated by real-time qRT-PCR, and both lnc-GAN1 and miR-26a-5p were enriched in the same precipitation by anti-AGO2 (*** *P* < 0.001). **g** Overexpressed miR-26a-5p promoted proliferation of H1299 cells, as shown by CCK8 assay. **h** Overexpressed miR-26a-5p accelerated cell cycle progression in H1299 cells. Data represent the mean ± SD of three independent experiments

Next, we wonder how the independently transcribed lnc-GAN1 exerts the tumor suppressor role in lung cancer. In general, the role or function of a biomolecule is mainly dependent on its location within a cell. Therefore, we first determined the subcellular distribution of this lncRNA in lung cancer cells. After extracting total RNA from the nucleus and cytoplasm of H1650 and H1299 cells, respectively, we performed qRT-PCR on the RNAs. In this assay, GAPDH and ACTB genes were employed as cytoplasmic positive controls, and U1 gene and LncRNA MALAT1 were used as nucleus positive control [30]. The results show that lnc-GAN1 is mostly detected in cytoplasmic RNA similar to the positive controls GAPDH and ACTB mRNAs (Fig. 5a left). Then we performed RNA FISH in H1650 and H1299 cells and NSCLC tumor tissues to visually observe the subcellular distribution of this lncRNA. Consistent with the result of qRT-PCR assay, we find that lnc-GAN1 is mainly located in the cytoplasm rather than in the nucleus in the two lung cancer cells (Fig. 5a right, and Additional file 2: Fig. S3) and NSCLC tissues (Additional file 2: Fig. S3), suggesting that lnc-GAN1 may act as a regulator at the post-transcriptional level in cytoplasm.

As we know, cytoplasmic lncRNA can act as a miRNA sponge in order to play its role in various cell processes [10]. Therefore, we assumed that lnc-GAN1 may function as a tumor suppressor via sponging and inhibiting miRNA. To this end, we searched 3 databases STARBASE (http://starbase.sysu.edu.cn/index.php), LncBase v.2 (https://diana.e-ce.uth.gr/tools), and PITAR (https://pictar.mdc-berlin.de) that predict the interaction between lncRNA and miRNA. All the 3 databases predict that lnc-GAN1 has the same seed sequence complementary with 3 miRNAs (Fig. 5b), including miR-26a-5p, mir-1297 and mir-26b-5p. To verify which miRNA interacts with lnc-GAN1, we carried out qRT-PCR on 30 pairs of NSCLC samples with the primers for the 3 miRNAs. The result shows that only miR-26a-5p has a negative correlation with lnc-GAN1 (*R*^2^ = − 0.45, *P* < 0.05, Fig. 5c). To further understand the interaction between lnc-GAN1 and miR-26a-5p, we overexpressed miR-26a-5p in H1299 and H1650 cells or lnc-GAN1 in A549 and H460 cells, respectively, and then observed the change in lnc-GAN1 or miR-26a-5p expression. Not unexpectedly, overexpressed miR-26a-5p significantly attenuates the expression of lnc-GAN1 in H1299 and H1650 cells (Fig. 5d); similarly, lnc-GAN1 overexpression also inhibits the expression of miR-26a-5p in A549 and H460 cells (Additional file 2: Fig. S4a), indicating that lnc-GAN1 and miR-26a-5p may directly interact with each other and that there is a negative correlation between their expressions in NSCLC cells.

Based on the public databases, lnc-GAN1 contains two seed sequences complementary to mir-26a-5p seed region (Fig. 5e upper-penal), implying that lnc-GAN1 may function as a sponge to inhibit miR-26a-5p. To confirm this point, we constructed lnc-GAN1 luciferase reporters that contained the putative wild-type or mutant miR-26a-5p binding sites (Fig. 5e upper-penal). As expected, miR-26a-5p overexpression significantly decreases the luciferase activity of the wild-type lnc-GAN1 reporter in H1299 and H1650 cells but did not affect that of the mutant lnc-GAN1 reporter (Fig. 5e lower-panel), indicating that lnc-GAN1 can directly bind to miR-26a-5p.

If lnc-GAN1 binds to miR-26a-5p, they will form a functional RNA-induced silencing complex (RISC) with AGO2 protein. Thus, we performed RIP experiments to pull-down the complex from H1650 cells using AGO2 antibodies and carried out qRT-PCR for lnc-GAN1 and miR-26a-5p in the pull-down complexes. The results reveal that lnc-GAN1 and miR-26a-5p are clearly enriched in the AGO2 antibody immunoprecipitated complexes compared with these in control IgG immunoprecipitation (Fig. 5f), demonstrating again that lnc-GAN1 can bind to miR-26a-5p in a structural and functional manner.

Finally, we want to know what role miR-26a-5p plays in NSCLC. To this purpose, we overexpressed miR-26a-5p in H1299 and H1650 cells and observed the change in cell proliferation, cell cycle, and apoptosis. The results show that miR-26a-5p overexpression promotes the proliferation and G2 phase progression and decreases apoptosis in both H1299 and H1650 cells (Fig. 5g, h and Additional file 2: Fig. S4b-e), suggesting that miR-26a-5p plays a oncogenic role in lung cancer cells. As mentioned above, we carried out qRT-PCR on mir-26a-5p in 30 pairs of NSCLC and adjacent normal lung tissues, whose expression is negatively correlated with lnc-GAN1 in NSCLC tissue. We also found that miR-26a-5p was highly expressed in the 30 NSCLC tissues compared with the matched normal lung tissues (Additional file 2: Fig. S4f), and tumors with a maximum diameter ≥ 3 cm have higher mir-26a-5p expression than the smaller tumors (< 3 cm) (Additional file 2: Fig. S4G), also implying the oncogenic role of miR-26a-5p in NSCLC tissues. These results suggest that lnc-GAN1 may function as a tumor suppressor via sponging miR-26a-5p and inhibiting its oncogenic role in NSCLC.

### PTEN is a direct target of miR-26a-5p and mediates the tumor-suppressive role of lnc-GAN1 in NSCLC

**Fig. 6:**
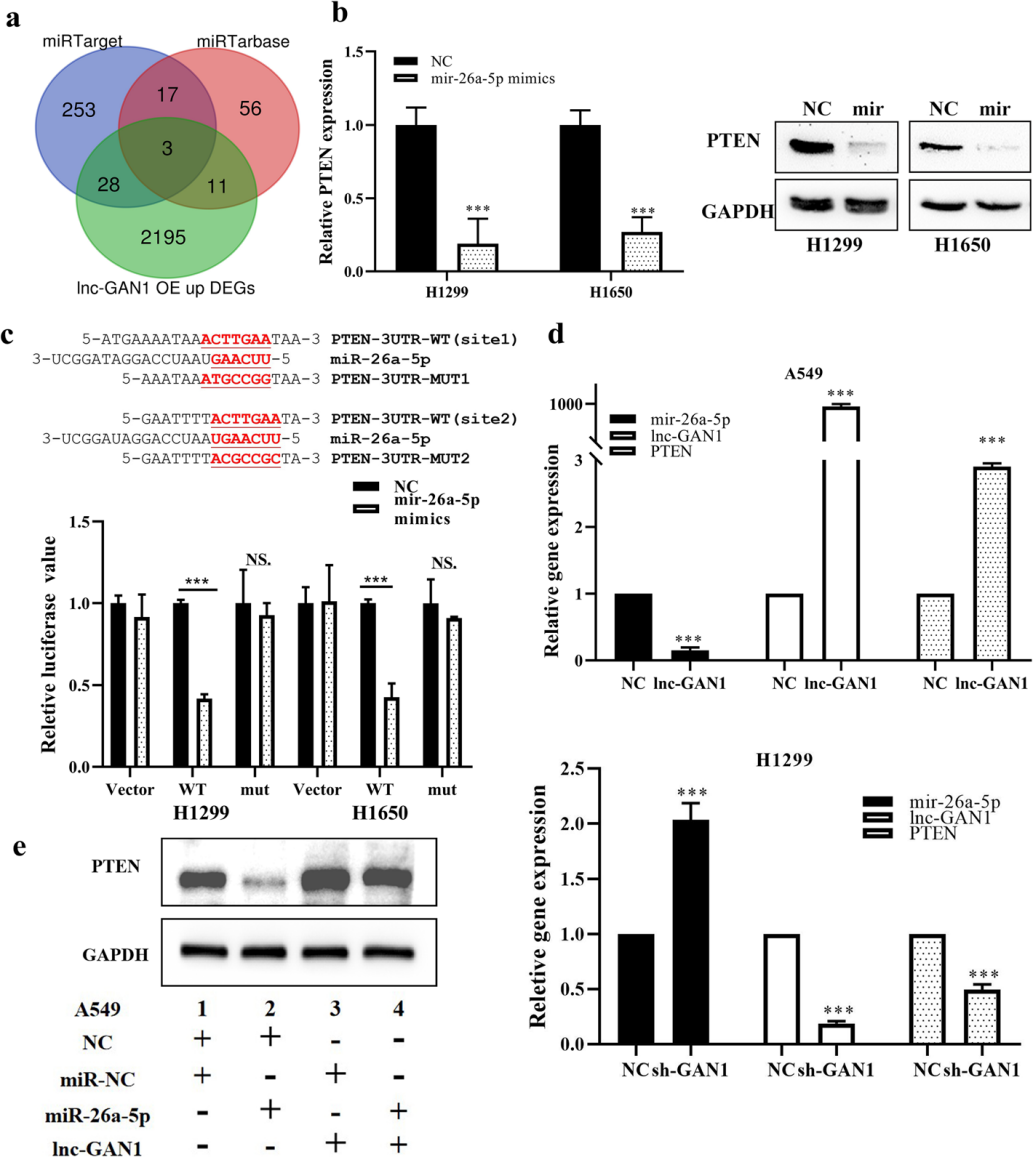
Lnc-GAN1 upregulates PTEN by inhibiting miR-26a-5p. **a** Venn diagram shows 3 shared predicted target genes of miR-26a-5p by two miRNA databases and overlapped with upregulated ones induced by expressed lnc-GAN1, including PTEN, CDK8, and PTGS2. **b** MiR-26a-5p overexpression reduces PTEN mRNA and protein levels in H1299 and H1650 cells (***P < 0.001), as determined by qRT-PCR and western blot. **c** Upper-Panel: The predicted wild-type seed sequences of PTEN 3’UTR complementary with miR-26a-5p and investigator-designed mutated seed sequences. Lower-panel: Histogram shows that when miR-26a-5p is overexpressed in H1299 and H1650 cells, the luciferase activity of the cells with wild-type seed sequence of PTEN 3’UTR is markedly reduced compared with that of control cells, but not changed in the cells with mutant miR-26a-5p binding sites, as assayed by Dual-luciferase reporter assays. **d** Lnc-GAN1 overexpression significantly decreases miR-26a-5p levels and increases PTEN mRNA levels in A549 cells, whereas lnc-GAN1 knockdown exerts the opposite effects in H1299 cells. **e** PTEN protein level is downregulated in A549 cells with miR-26a-5p overexpression, upregulated in the cells with lnc-GAN1 expression and not changed in the cells co-transfected with lnc-GAN1 and miR-26a-5p

In the above experiments, we demonstrated that lnc-GAN1 functioned as a sponge to directly inhibit miR-26a-5p in NSCLC, which will lead to upregulation of the target genes of miR-26a-5p to mediate the tumor-suppressive effects of lnc-GAN1. Therefore, we want to know which target gene(s) of miR-26a-5p mediates the tumor suppressor role of lnc-GAN1 in lung cancer. To this end, we first profiled gene expression in A549 cells overexpressing lnc-GAN1 and control cells to find the upregulated genes that are also known to be the targets of miR-26a-5p in the public databases. The result showed that there were 3 upregulated genes (CDK8, PTEN, and PTGS2) in A549 cells with lnc-GAN1 overexpression, which are also the predictive targets of miR-26a-5p in miRTarget and miRTarbase databases (Fig. 6a). Of the three upregulated genes, CDK8 and PTGS2 have been reported to be oncogenic gene [31, 32], which cannot mediate the tumor-suppressor role; PTEN is one of the most important tumor suppressor genes in cancers, and meets both requirements: a direct target of miR-26a-5p and a possible indirect downstream gene of the tumor-suppressive lnc-GAN1. To verified which gene(s) can be targeted by miR-26a-5p, we overexpressed miR-26a-5p in H1299 cells and found that only PTEN expression was decreased and CDK8 and PTGS2 expression did not change in H1299 cells (Additional file 2: Fig. S5a), implying that only PTEN may be a target of miR-26a-5p in lung cancer. Thus, PTEN was selected for further study.

Then, we want to verify that PTEN is a direct target of miR-26a-5p. To this purpose, we firstly transfected H1299 and H1650 cells with miR-26a-5p mimics and then observed the change of PTEN expression in the two cells. The result indicates that ectopic expression of miR-26a-5p indeed downregulated PTEN mRNA and protein levels in H1299 and H1650 cells (Fig. 6b). We next attempt to determine whether PTEN could be directly bound by miR-26a-5p. Thus, we inserted wild-type or mutant PTEN 3′UTR sequence (Fig. 6c upper-penal) into downstream of a luciferase reporter vector, and co-transfected the recombinant luciferase reporter vectors and miR-26a-5p mimics or scramble controls into H1299 and H1650 cells. As expected, miR-26a-5p overexpression reduced the luciferase activity of the wild-type PTEN 3′UTR reporter, but had no effect on the mutant reporter in the two cell lines (Fig. 6c lower-panel), indicating that miR-26a-5p can directly bind to 3’UTR of PTEN gene.

Next, we want to figure out whether lnc-GAN1 can induce PTEN expression by inhibiting miR-26a-5p in lung cancer cells. To this end, we detected miR-26a-5p and PTEN expression in H1299 and A549 cells with downregulation or overexpression of lnc-GAN1, respectively. The results demonstrate that ectopic lnc-GAN1 expression upregulates PTEN mRNA and downregulates mir-26a-5p in A549 cells (Fig. 6d upper-penal), while lnc-GAN1 knockdown exerted the opposite effects in H1299 cells (Fig. 6d lower-panel), suggesting that the tumor-suppressor role of lnc-GAN1 is mediated by PTEN via this lncRNAs’ sponging and inhibiting miR-26a-5p.

**Fig. 7:**
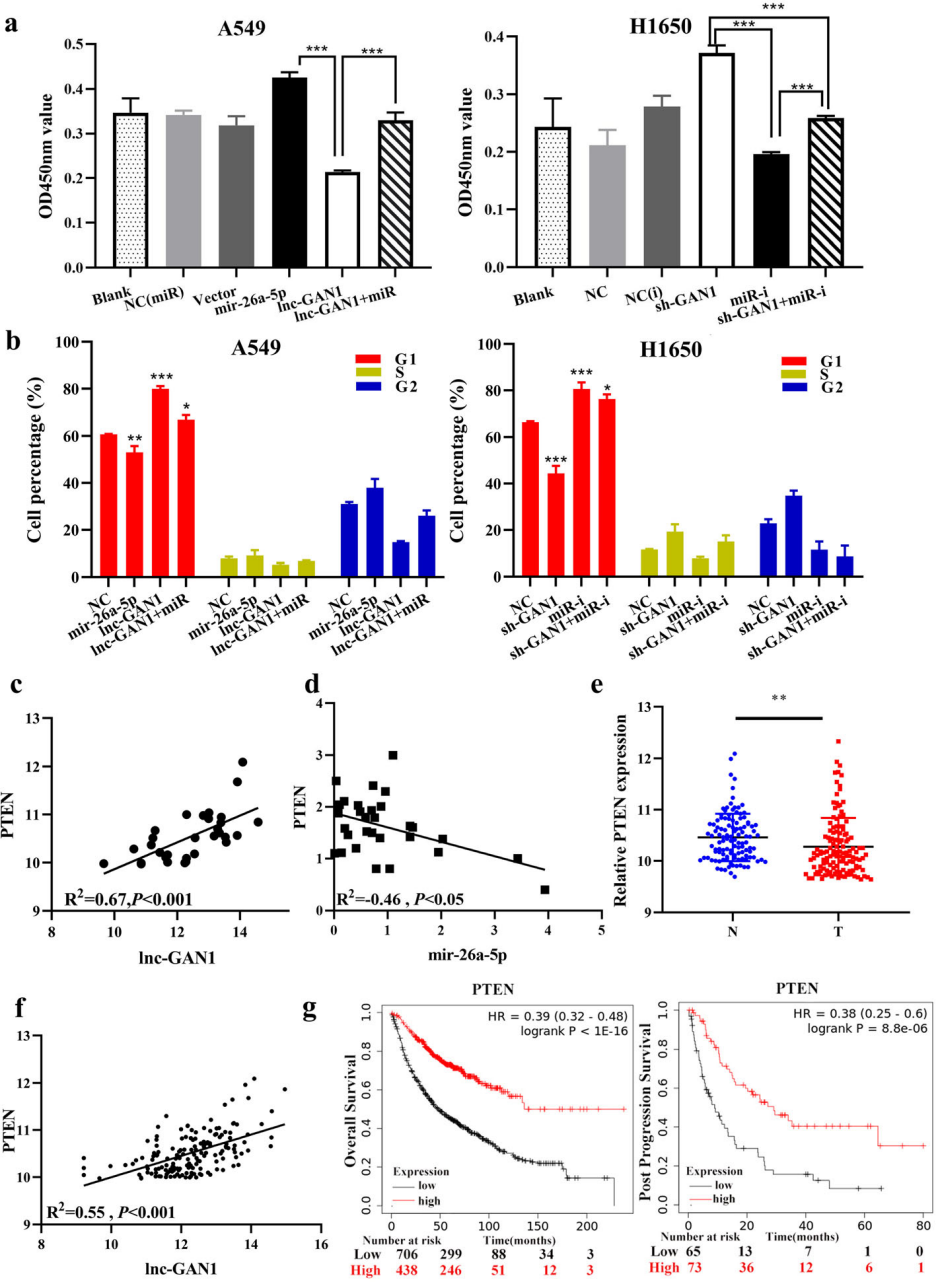
lnc-GAN1 inhibits NSCLC progression by repressing miR-26a-5p to activate PTEN signaling. **a** miR-26a-5p overexpression alone markedly increases the cell proliferation and lnc-GAN1 overexpression alone remarkably decreases cell proliferation in A549 cells compared with blank or respective control treatments, whereas when both are overexpressed, cell proliferation is not changed; downregulation of miR-26a-5p alone or lnc-GAN1 alone or both combination produces opposite effects on cell proliferation in H1650 cells. **b** miR-26a-5p overexpression alone significantly promotes cell cycle progression and lnc-GAN1 overexpression alone has the reverse effect on cell cycle, whereas when both are overexpressed, the cell cycle is not changed; downregulation of miR-26a-5p alone or lnc-GAN1 alone or both combination produces opposite effects on cell cycle in H1650 cells. **c** There is a positive correlation between lnc-GAN1 and PTEN levels in 30 NSCLC tissues, as measured by qRT-PCR (*R*^2^ = 0.55, *P* < 0.05). **d** There is a negative correlation between PTEN and miR-26a-5p levels in 30 NSCLC tissues, as determined by qRT-PCR (*R*^2^ = − 0.46, *P* < 0.05). **e** PTEN mRNA levels in 194 NSCLC samples are significantly higher than those in 100 normal lung tissues, as measured by our custom microarray. **f** In TCGA database, PTEN mRNA is significantly decreased in 515 lung adenocarcinomas compared with that in 51 normal tissues (*P* < 0.001). **g** In TCGA database, NSCLC patients with high PTEN expression have significantly better overall survival (left, *P* < 0.001) and post progression survival (right, *P* < 0.001) than those with low PTEN expression

To verify the role of the lnc-GAN1/miR-26-5p/PTEN signaling in lung cancer cells, we carried out rescue experiments on lung cancer cells. The results suggest that miR-26a-5p overexpression can not only reduce endogenous PTEN protein but also reverse the upregulation of PTEN protein induced by lnc-GAN1 in A549 cells (Fig. 6e); CCK8 and cell cycle assays revealed that miR-26a-5p overexpression can not only promote cell proliferation and G1-phase cell cycle progression but also restore the cell proliferation and G1-phase cell cycle progression that are inhibited by lnc-GAN1 in A549 and H1650 cells (Fig. 7a, b). Similar results were also obtained in H460 and H1299 cells (Additional file 2: Fig. S5b-c). These data confirm that lnc-GAN1 plays a tumor suppressor role by activating lnc-GAN1/miR-26-5p/ PTEN signaling in NSCLC.

Finally, to verify the interaction among lnc-GAN1, miR-26a-5p and PTEN in NSCLC tissues, we investigated PTEN expression with qRT-PCR in the 30 NSCLC tissues that already have been detected for lnc-GAN1 and miR-26a-5p. The result showed that PTEN expression positively correlated with lnc-GAN1 (Fig. 7c, *R*^2^ = 0.67, *P* < 0.001) and negatively correlated with miR-26a-5p in NSCLC tissues (Fig. 7d, *R*^2^ = − 0.46, *P* < 0.05), indicating that PTEN is a target gene of miR-26a-5p and mediates the tumor-suppressor role of lnc-GAN1 in NSCLC. Furthermore, in our lncRNA microarray that had, coincidentally, a probe for PTEN mRNA, we simultaneously analyzed the expressions of lnc-GAN1 and PTEN and their relationship. The result exhibits that PTEN expression is downregulated in NSCLC tissues (Fig. 7e) and positively correlated with lnc-GAN1 expression (Fig. 7f). TCGA data also reveal that PTEN is downregulated in NSCLC tissues including lung adenocarcinoma (Additional file 2: Fig. S5d) and lung squamous cell carcinoma (Additional file 2: Fig. S5e) compared with that in normal lung tissues, and correlated with poor OS and post progression survival (PPS) of NSCLC patients (Fig. 7g). Altogether, these results suggest that lnc-GAN1 upregulates and activates PTEN by sponging and inhibiting miR-26a-5p in NSCLC, leading to G1-phase cell cycle arrest.

### Lnc-GAN1 inhibits progression of NSCLC via regulating the miR-26a-5p/PTEN signaling

**Fig. 8:**
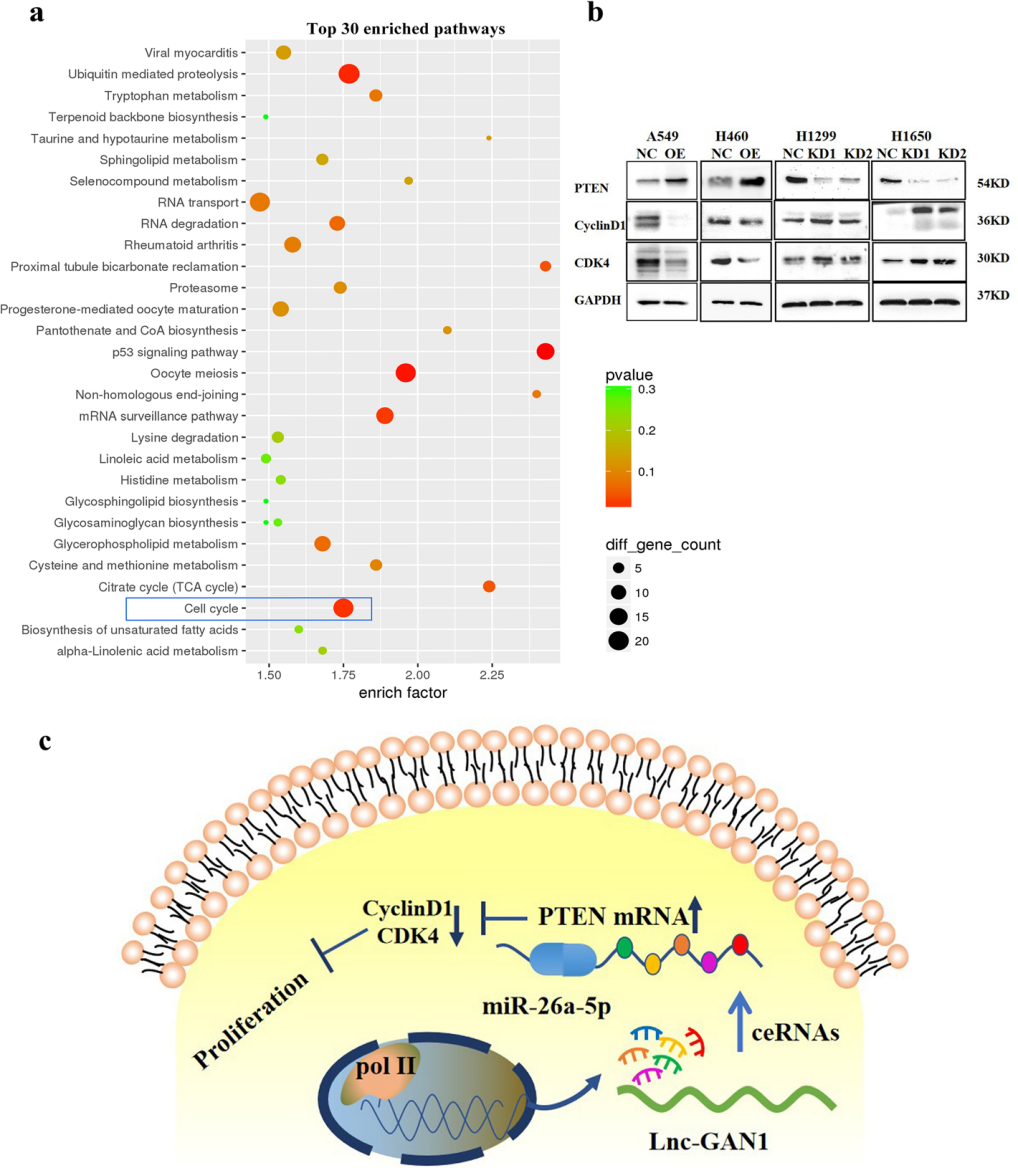
Lnc-GAN1 repressed cell proliferation through cell cycle signaling pathway. **a** KEGG pathway analysis of differentially expressed genes that were generated by the SurePrint G3 Human Microarray analysis of A549-NC and A549-lnc-GAN1 stable cells indicates that cell cycle signaling pathway is involved in lnc-GAN1 inhibiting NSCLC progression. **b** Overexpressed lnc-GAN1 elevates PTEN protein and decreases Cyclin D1 and CDK4 proteins in A549 and H460 cells, while lnc-GAN1 downregulation has the opposite effects in H1299 and H1650 cells. **c** Molecular mechanism of lnc-GAN1

In the aforementioned experiment, we profiled gene expression of A549 cells with lnc-GAN1 overexpression. To explore which cell signaling pathway is regulated by the lnc-GAN1/miR-26-5p/PTEN axis, we utilized the gene expression profile data to perform KEGG pathway and protein-protein interaction network (PPIN) analysis. KEGG pathway analysis indicated that lnc-GAN1/miR-26-5p/PTEN axis was involved in cell cycle signaling pathway (Fig. 8a), and PPIN analysis also suggested that PTEN was a key gene related to the cell cycle signaling pathway (Additional file 2: Fig. S5f), which is consistent with the reported findings that the activated PTEN induces G1-phase cell cycle arrest [33]; we also confirmed the G1-phase arrest caused by activated lnc-GAN1/miR-26-5p/PTEN axis in the above experiment.

To corroborate that lnc-GAN1/miR-26-5p/PTEN axis regulates cell cycle signaling pathway, we employed western blot to detect key cyclins of cell cycle signaling in NSCLC cells with lnc-GAN1 overexpression or downregulation. The results confirmed that the activated lnc-GAN1/miR-26-5p/ PTEN signaling axis markedly increased PTEN protein level and sharply reduced Cyclin D1 and CDK4 protein levels, which restrained cell cycle progression in A549 and H460 cells with lnc-GAN1 overexpression (Fig. 8b left), whereas the inactivated signaling axis produced the opposite effects in H1299 and H1650 cells with lnc-GAN1 downregulation (Fig. 8b right). According to reported studies, Cyclin D1 and CDK4 form a complex to promote cell cycle progression. Since enhanced PTEN expression and downregulated Cyclin D1 and CDK4 were observed in the same cells, we wonder whether PTEN can directly bind to Cyclin D1/CDK4 complexes. Thus, we performed a co-immunoprecipitation with antibody against PTEN and western blot on H1650 cells with lnc-GAN1 overexpression. As shown in Additional file 2: Fig. S5g, PTEN can bind to Cyclin D1 and CDK4 protein, indicating that PTEN can bind to and inhibit Cyclin D1/CDK4 complexes. Taken together, lnc-GAN1 represses the progression of NSCLC by sponging and inhibiting miR-26a-5p to activate PTEN signaling pathway (Fig. 8c).

## Discussion

NSCLC, which accounts for approximately 85% of all lung cancer cases, is characterized by rapid progression, a high mortality rate, and a low five-year survival rate [34, 35]. In recent years, accumulating evidence has indicated that lncRNAs play an important role in regulating gene expression at the epigenetic, transcriptional, post-transcriptional, translational and post-translational levels, which are in involved in diverse diseases and cancers including NSCLC tumorigenesis and progression [36, 37]. LncRNAs harboring microRNA binding sites can act as sponges for miRNAs and thus post-transcriptionally regulate the expression and biological activity of miRNA target genes via their ability to compete for miRNA binding to the 3’UTR of target mRNAs. For instance, lncRNA-PVT1 competitively binds to miR-424-5p to regulate CARM1 expression and reduce the sensitivity of NSCLC to radiotherapy [38]. However, LINC01436 functions as a miR-30a-3p sponge to regulate the expression of miR-30a-3p target gene EPAS1, thus promoting NSCLC growth and metastasis [39]. Moreover, LINC 00673 can sponge miR-150-5p to upregulate the mRNA and protein levels of ZEB1, a key epithelial-mesenchymal transition regulator, thereby acting as an oncogenic lncRNA in NSCLC [17].

In this study, we demonstrate for the first time that lnc-GAN1 functions as an endogenous sponge for miR-26a-5p to upregulate PTEN expression and thereby exert tumor-suppressive effects in NSCLC. First, we find that lnc-GAN1 is significantly downregulated in NSCLC tissues by using a custom lncRNA microarray on 194 NSCLC tissues and 100 paired adjacent non-tumor lung samples. Kaplan-Meier survival analysis reveals that lnc-GAN1 downregulation is associated with poor prognosis in these patients. In addition, functional studies demonstrate that lnc-GAN1 inhibits NSCLC cell proliferation and cell cycle and induces apoptosis in vitro, and represses tumor growth in vivo.

By searching PubMed, we found no report on lnc-GAN1.. lnc-GAN1 is located in 3′UTR of the GAN gene on chromosome 16. However, we demonstrate that transcription of lnc-GAN1 is independent of the GAN gene expression. The 3′UTR of a gene is involved in the stability, translation, translocation, and transport of its mRNA. Moreover, recent studies have shown that 3′UTR or its cleaved fragments can be expressed independently of the parental gene and act as long non-coding or small RNAs. Although some lncRNAs derived from 3’UTR have been identified, their biological functions remain unclear [21, 23, 24, 40].

Furthermore, RNA FISH assay revealed that lnc-GAN1 is mainly located in the cytoplasm of NSCLC cell lines and tissue samples, suggesting that it may function as a miRNA sponge. Bioinformatics analysis showed that there are two predicted binding sites for miR-26a-5p in lnc-GAN1 sequence. Dual-luciferase reporter assay confirmed the direct interaction between lnc-GAN1 and miR-26a-5p, while qRT-PCR demonstrated that lnc-GAN1 and miR-26a-5p levels are inversely correlated in NSCLC samples. In addition, miR-26a-5p overexpression significantly reduced lnc-GAN1 level in NSCLC cell lines, further supporting the role of lnc-GAN1 as an endogenous sponge for miR-26a-5p.

MiR-26a-5p has previously been reported to promote cell proliferation, accelerate G1/S cell cycle transition, and enhance cancer metastasis [41–43]. Moreover, miR-26a-5p upregulation predicts poor prognosis in lung cancer and has been proposed to be used as a potential prognostic biomarker [44]. In this study, miR-26a-5p was highly expressed in patients with NSCLC, particularly those with large tumors. In vitro experiment shows that miR-26a-5p overexpression promotes NSCLC cell proliferation and inhibits apoptosis, thereby directly opposing the effects of lnc-GAN1. Thus, lnc-GAN1 exerts its tumor suppressor role in NSCLC cells by competing with miR-26a-5p to upregulate the target genes.

Previous studies have validated PTEN as a direct target of miR-26a-5p and showed that miR-26a-5p promotes cancer progression by down-regulating PTEN and subsequently activating the downstream PI3K/AKT pathway [43]. We confirm the negative correlation between mir-26a-5p and PTEN levels in NSCLC clinical samples in this study and find that miR-26a-5p overexpression reduces the mRNA and protein levels of PTEN in NSCLC cell lines. Dual-luciferase reporter assays further revealed that miR-26a-5p can specifically bind to the 3′UTR region of PTEN.

PTEN is a well-known tumor suppressor and negative regulator of the PI3K/AKT signaling, which is frequently altered in various cancers [45, 46]. Reduced PTEN protein levels have been detected in the majority of lung cancers [47]; however, genetic alterations in the PTEN gene are rare [48]. Here we confirm that PTEN mRNA level is reduced in NSCLC tissues compared with those in the corresponding healthy lung tissues, and PTEN mRNA level is positively correlated with lnc-GAN1 levels in NSCLC tissues. In addition, lnc-GAN1 overexpression significantly downregulates miR-26a-5p and upregulated PTEN mRNA levels in H1299 and H1650 cells, whereas lnc-GAN1 knockdown exerted the opposite effect. These findings support the hypothesis that lnc-GAN1 functions as an endogenous miR-26a-5p sponge to upregulate PTEN expression.

Previous studies have shown that a few lncRNAs can regulate PTEN mRNA levels by competitively binding to miRNAs. For instance, lncRNA-AC078883.3 has been reported to regulate the PTEN/AKT signaling pathway by sponging miR-19a, thus suppressing the development of cisplatin chemoresistance in lung cancer [40]. Similarly, lncRNA TP53TG1 has been shown to enhance the sensitivity of NSCLC cells to cisplatin by modulating the miR-18a/PTEN axis [48, 49]. To elucidate whether lnc-GAN1 acted via the miR-26a-5p/PTEN pathway in NSCLC, we performed RNA-seq in lnc-GAN1 overexpressing cells and found that lnc-GAN1 significantly affected the expression of cell-cycle-related genes, such as PTEN. It has been reported that PTEN mediates tumor suppression by negatively regulating the PI3K/AKT signaling pathway, which controls and coordinates two major cellular processes, cell cycle progression and cell death [50]. We validated the interaction between PTEN and the cell-cycle-related proteins CDK4 and Cyclin D1 with Co-IP and revealed that lnc-GAN1 overexpression increased PTEN level and decreased CDK4 and cyclin D1 levels in NSCLC cell lines, whereas lnc-GAN1 knockdown had the opposite effects. In addition, miR-26a-5p overexpression could rescue the effects of lnc-GAN1 overexpression on cell proliferation and cell cycle. Taken together, our findings show that lnc-GAN1 regulates PTEN by functioning as an mRNA sponge.

## Conclusions

We identified that lnc-GAN1 was downregulated in NSCLC tissues. Moreover, reduced lnc-GAN1 levels were associated with large tumors and poor prognosis in patients with NSCLC. This lncRNA functions as a miR-26a-5p sponge to upregulate PTEN mRNA level, thereby suppressing cell proliferation and inducing apoptosis in NSCLC. Our study highlights the crosstalk between lnc-GAN1 and the miR-26a-5p/PTEN axis and suggests that lnc-GAN1 may serve as a potential novel prognostic biomarker and therapeutic target in NSCLC patients.

## Supplementary Information

**Additional file 1: Supplementary Table S1.** Sequences of primers and siRNA used in this study. **Supplementary Table S2.** Univariate and multivariate Cox regression analyses of factors associated with overall survival in patients with NSCLC.

**Additional file 2: Supplementary Figure S1.** Tumor suppressor role of Lnc-GAN1 in NSCLC cells. **Supplementary Figure S2.** Location and predicted promoters of lnc-GAN1. **Supplementary Figure S3.** Lnc-GAN1 was located in cytoplasm. **Supplementary Figure S4.** Cell biological functions of MiR-26a-5p in NSCLC. **Supplementary Figure S5.** miR-26a-5p promotes proliferation, cell cycle and apoptosis of NSCLC cells. **Supplementary Figure S6.** Lnc-GAN1 represses oncogenic phenotype of NSCLC cells by sponging and inhibiting miR-26a-5p to activate PTEN signaling.

## Data Availability

All data and materials in this study are available upon request.

## Abbreviations

OS: Overall survival
PPS: Post progression survival
FISH: Fluorescence in situ hybridization
PPI: Protein-protein interaction
RIP: RNA immunoprecipitation
Co-IP: Co-immunoprecipitation
NSCLC: Non-small-cell lung cancer
LC: Lung cancer
PBS: Phosphate buffered saline
PTEN: Phosphatase and tensin homolog
CDK8: Cyclin-dependent kinase 8
PTGS2: Prostaglandin-Endoperoxide Synthase 2
