# Supplementary material for: Lnc-GAN1 expression is associated with good survival and suppresses tumor progression by sponging mir-26a-5p to activate PTEN signaling in non-small cell lung cancer
Source: J Exp Clin Cancer Res. 2021 Jan 6;40:9. doi: 10.1186/s13046-020-01819-0 (PMC7786923; doi:10.1186/s13046-020-01819-0)
Supplement: Supplementary file 1 — Additional file 1: Supplementary Table S1. Sequences of primers and siRNA used in this study. Supplementary Table S2. Univariate and multivariate Cox regression analyses of factors associated with overall survival in patients with NSCLC. [file 13046_2020_1819_MOESM1_ESM.docx]

**Additional File 1: Supplementary Tables**

**Lnc-GAN1 expression is associated with good survival and suppresses tumor progression by sponging mir-26a-5p to activating PTEN signaling in non-small cell lung cancer**

Rui-Qi Wang^1,5^, et al

1. Supplementary Table S1
2. Supplementary Table S2

**Additional File 1**

**Table 1. Primers and siRNA used in this study**

| **primer** | **sequence** |
| --- | --- |
| GAPDH-homo sapien (F) | 5'-GAAATCCCATCACCATCTTCCAGG-3' |
| GAPDH-homo sapien(R) | 5'-GAGCCCCAGCCTTCTCCATG-3' |
| PTEN(F) | 5'-TCCATCCTGCAGAAGAAGCC-3' |
| PTEN(R) | 5'-CTGTCATGTCTGGGAGCCTG-3' |
| RNU1-1-homo sapien (F) | 5'-TACTTACCTGGCAGGGGAGATAC-3' |
| RNU1-1-homo sapien (R) | 5'-GAACGCAGTCCCCCACTAC-3' |
| LncGAN1(F) | 5'-GGGCAACAAGGGCTAAAC-3' |
| LncGAN1(R) | 5'-AAGGCTACCCACCTACGG-3' |
| GAN1_ChIP(F) | 5'-CACTTACAACCAGGAGGAA-3' |
| GAN1_ChIP(R) | 5'-TCAGATGACAGATAGACACA-3' |
| PTGS2-F | 5'-TGAGCATCTACGGTTTGCTG-3' |
| PTGS2-R | 5'-TGCTTGTCTGGAACAACTGC-3' |
| CDK8-F | 5'-TGCCCTCCCTCCTCCTCTCTTT-3' |
| CDK8-R | 5'-TCACGCCTCACGCTCTCACA-3' |
| hsa-miR-26a-5p (qF) | 5'-TCGGCAGGTTCAAGTAATCCAG-3' |
| hsa-miR-26a-5p (qR) | 5'-CAGTGCGTGTCGTGGAGT-3' |
| MALAT1（F） | 5'-CTTCCCTAGGGGATTTCAGG-3' |
| MALAT1(R) | 5'-GCCCACAGGAACAAGTCCTA-3' |
| si-GAN1-Homo-158 F | 5'-GGAAGUAGAAGUAGGAUUUTT-3' |
| si-GAN1-Homo-158 R | 5'-AAAUCCUACUUCUACUUCCTT-3' |
| si-GAN1-Homo-270 F | 5'-GCAGCAUCCUGUUUGACAUTT-3' |
| si-GAN1-Homo-270 R | 5'-AUGUCAAACAGGAUGCUGCTT-3' |

**Table S2. Univariate and** **multivariate Cox regression analyses of factors associated with overall survival in patients with NSCLC**

|  | **Univariate analysis** | | | **Multivariate analysis** |  |  |
| --- | --- | --- | --- | --- | --- | --- |
| **Characteristics** | ***P* value** | **HR** | **95% CI** | ***P* value** | **HR** | **95% CI** |
| **Age**(≥60 vs <60) | 0.215 | 1.233 | 0.885—1.718 |  |  |  |
| **Gender**  (Male vs. Female) | 0.129 | 0.742 | 0.505—1.09 |  |  |  |
| **Clinical Stage**  (I/II vs. III/IV) | **0.004** | 0.578 | 0.400—0.836 | **0.020** | 0.540 | 0.321—0.908 |
| **Tumor size**  (<3cm vs. ≥3cm) | **0.042** | 0.670 | 0.498—1.035 | 0.201 | 1.477 | 0.812—2.688 |
| **Histological tumor type**  (ADC/SCC) | 0.800 | 1.044 | 0.749—1.454 |  |  |  |
| **Lymph node metastasis**  (Yes vs. No） | 0.459 | 0.882 | 0.632—1.231 |  |  |  |
| **Metastasis**  (Yes vs. No） | 0.324 | 0.749 | 0.421—1.331 |  |  |  |
| **Smoking history**  (Yes vs. No） | 0.088 | 1.334 | 0.958—1.858 |  |  |  |
| **Lnc-GAN1**  (High vs. Low) | **0.002** | 0.566 | 0.395—0.810 | **0.009** | 0.572 | 0.376—0.873 |
| * **CI: confidence interval** | | | |  |  |  |
