# Supplementary material for: Lnc-GAN1 expression is associated with good survival and suppresses tumor progression by sponging mir-26a-5p to activate PTEN signaling in non-small cell lung cancer
Source: J Exp Clin Cancer Res. 2021 Jan 6;40:9. doi: 10.1186/s13046-020-01819-0 (PMC7786923; doi:10.1186/s13046-020-01819-0)
Supplement: Supplementary file 2 — Additional file 2: Supplementary Figure S1. Tumor suppressor role of Lnc-GAN1 in NSCLC cells. Supplementary Figure S2. Location and predicted promoters of lnc-GAN1. Supplementary Figure S3. Lnc-GAN1 was located in cytoplasm. Supplementary Figure S4. Cell biological functions of MiR-26a-5p in NSCLC. Supplementary Figure S5. miR-26a-5p promotes proliferation, cell cycle and apoptosis of NSCLC cells. Supplementary Figure S6. Lnc-GAN1 represses oncogenic phenotype of NSCLC cells by sponging and inhibiting miR-26a-5p to activate PTEN signaling. [file 13046_2020_1819_MOESM2_ESM.docx]

**Additional File 2: Supplementary Figures**

**Lnc-GAN1 expression is associated with good survival and suppresses tumor progression by sponging mir-26a-5p to activating PTEN signaling in non-small cell lung cancer**

Rui-Qi Wang^1,5^, et al

1. Supplementary Figure S1
2. Supplementary Figure S2
3. Supplementary Figure S3
4. Supplementary Figure S4
5. Supplementary Figure S5

**Additional File 2**


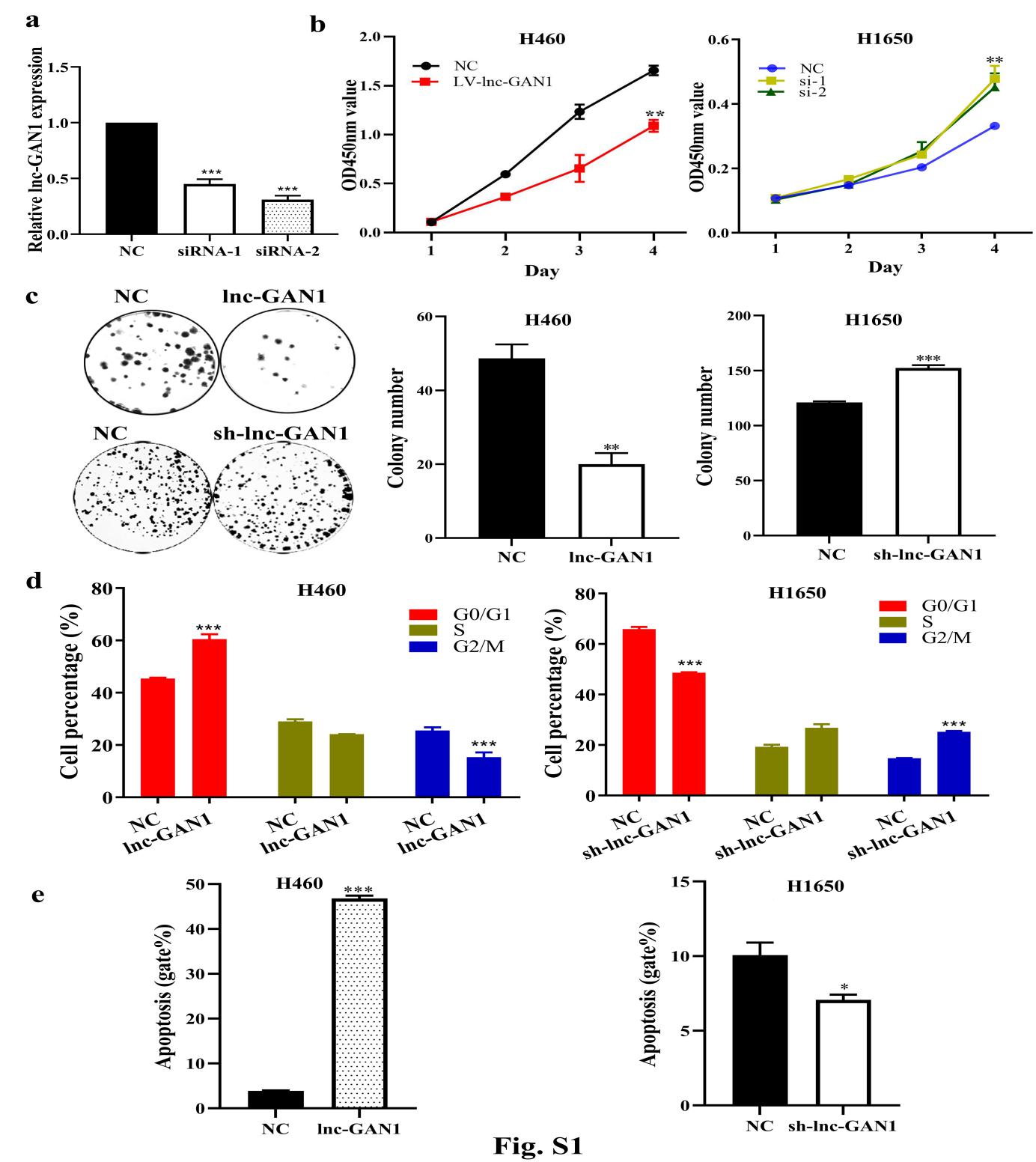


**Figure S1. Tumor suppressor role of Lnc-GAN1 in NSCLC cells.** (a) Lnc-GAN1 expression was significantly downregulated by transfection of siRNAs against lnc-GAN1 in H1650 cells. (b) lnc-GAN1 overexpression markedly reduces colony formation compared with control vector in H460 cells (P < 0.010); lnc-GAN1 downregulation by shRNA enhances colony formation compared with control vector in H1650 stable cells. (**c**) lnc-GAN1 overexpression remarkably inhibits cell proliferation compared with control vector in H460 cells (P < 0.010); lnc-GAN1 downregulation by siRNA has the opposite effect on cell proliferation in H1650 cells, as determined by CCK8 assay. (**d**) Lnc-GAN1 overexpression arrests cell cycle at G1 phase compared with control vector in H460 cells (left), and lnc-GAN1 downregulation by shRNA has the reverse effect on cell cycle in H1650 cells (right), as detected by flow cytometry (left). Histograms represent the percentage of cells in G0/G1, S, or G2/M phase. Data represent mean ± SD of three independent experiments (**P* < 0.05; ****P* < 0.001, by Student’s *t*-test). (**e**) Histograms show that Lnc-GAN1 overexpression induces significant apoptosis compared with control vector in H460 cells (left), and lnc-GAN1 downregulation by shRNA has the reverse effect on apoptosis in H1650 cells (right), as detected by Flow cytometry assays.





**Figure S2. Promoter prediction and Location of lnc-GAN1 in 3'UTR of its parent gene GAN.** (a) Lnc-GAN1 was located in 3`UTR of its parent gene GAN. (**b**) Four promoter sequence regions of lnc-GAN1 in eukaryotic sequence were predicted (cut-off =0.90) by Neural Network Promoter Prediction program. (**c**) The designed primers cover3 predicted promoter regions (943-993, 980-1030, 1000-1050 bp) of lnc-GAN1 for ChIP-PCR.


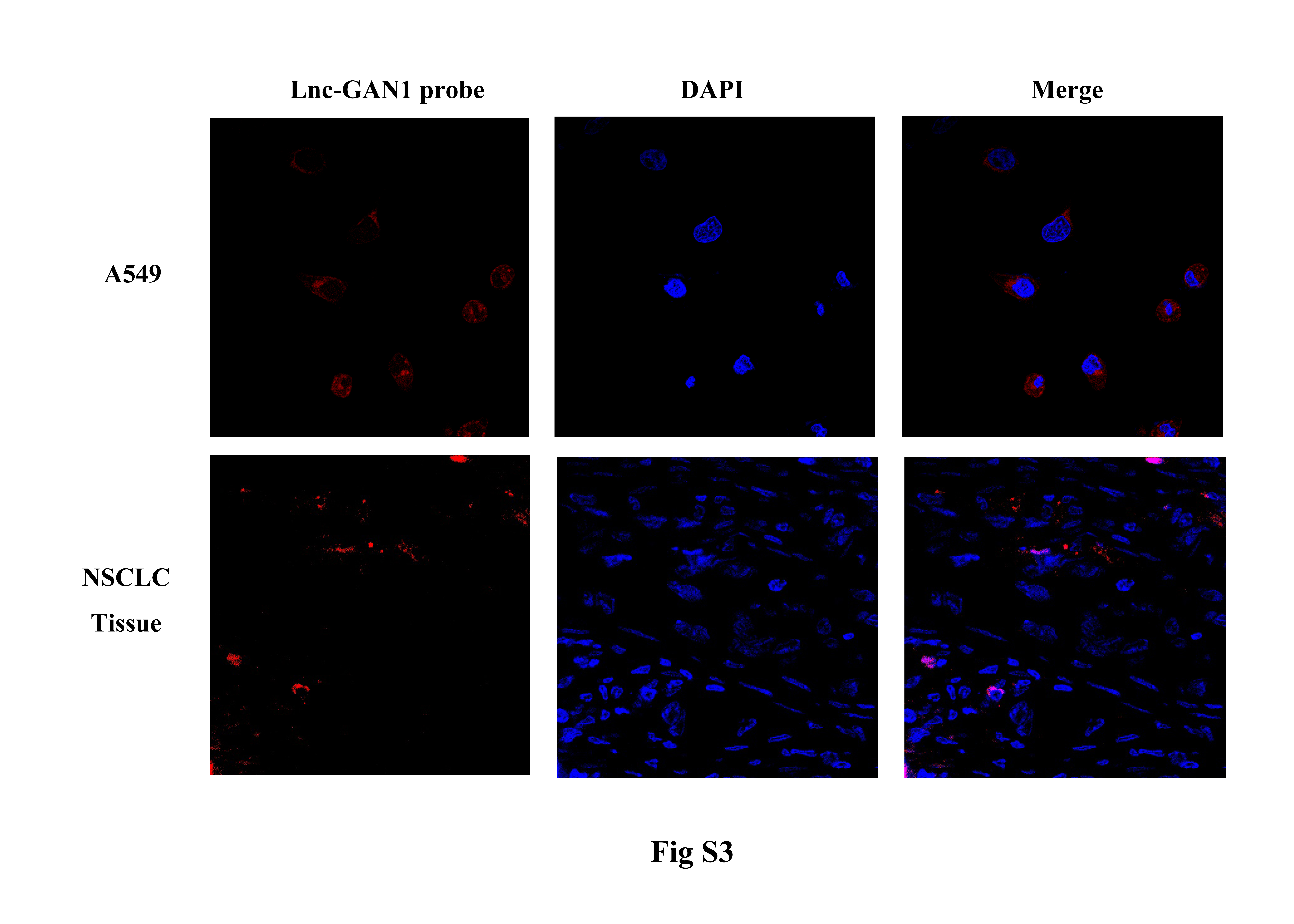


**Figure S3. Lnc-GAN1 was located in cytoplasm.** Lnc-GAN1 is located in the cytoplasm of NSCLC cells (A549 cells, upper-panel) and tissues (lower-panel), as determined by FISH.


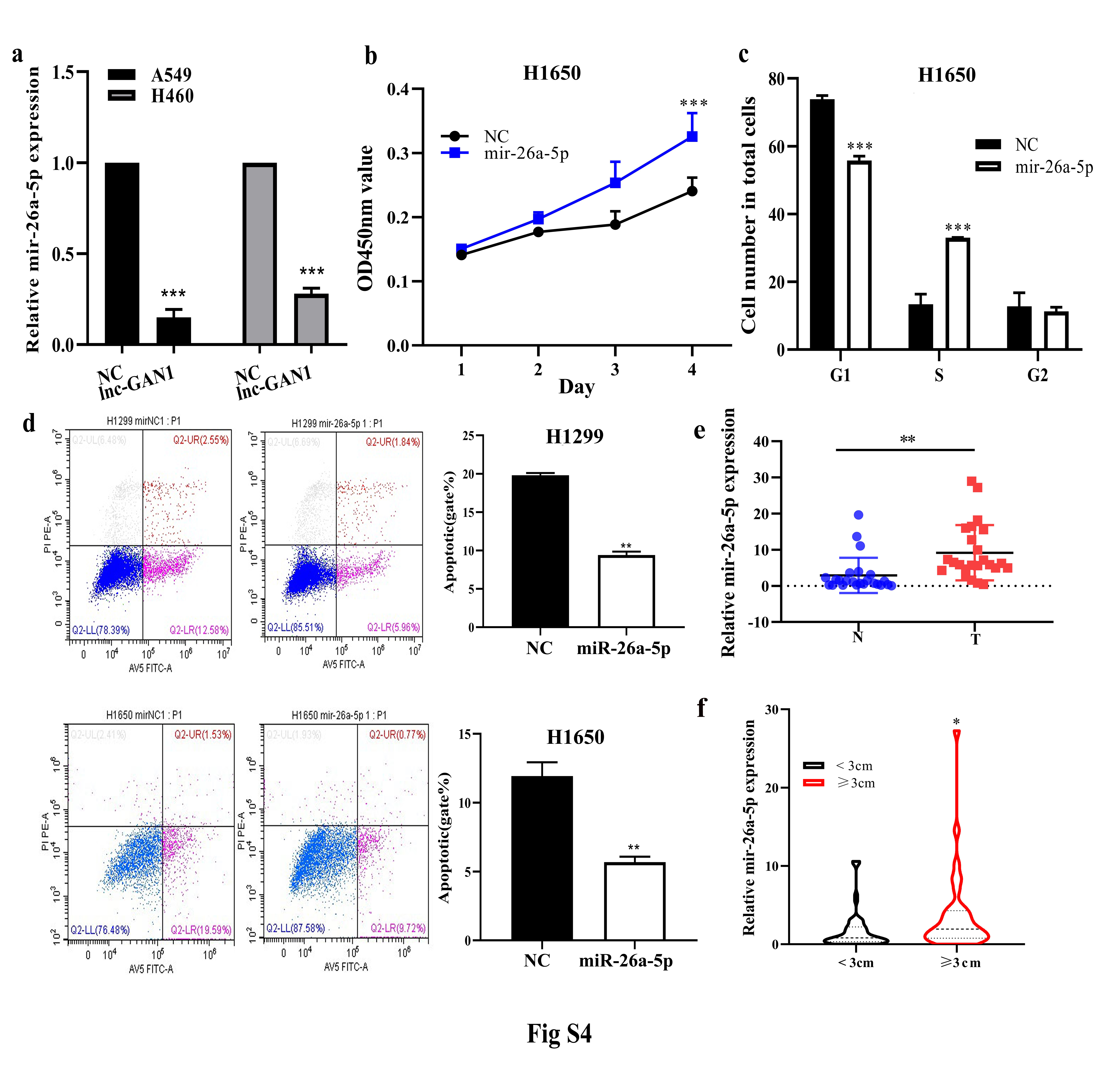


**Figure S4.** **MiR-26a-5p promotes proliferation, cell cycle and apoptosis of NSCLC cells.** (a) Overexpressed lnc-GAN1 reduces expression of miR-26a-5p in A549 and H460 cells, as measured by qRT-PCR. (**b**) miR-26a-5p overexpression promotes the proliferation of H1650 cells compared with control vectors, as detected by CCK8 assay. (**c**) Ectopic expression of mir-26a-5p accelerated G1/S phase transition in H1650 cells compared with control vector, as detected by flow cytometry. (**d**-**e**) Mir-26a-5p overexpression inhibits apoptosis of H1299 and H1650 cells, as determined by flow cytometry. **(f)** MiR-26a-5p level is higher in NSCLC tissues than that in the adjacent lung tissues (**P* < 0.01). **(g)** The expression level of mir-26a-5p is higher in lung cancer tissues larger than 3 cm than those less than 3 cm. (***P* < 0.01).





**Figure S5.** **Lnc-GAN1 represses oncogenic phenotype of NSCLC cells by sponging and inhibiting miR-26a-5p to activate PTEN signaling.** (a) qRT-PCR analysis shows that miR-26a-5p overexpression only inhibits PTEN expression but not PSTG2 and CDK8 in H1299 cells. (**b**) miR-26a-5p overexpression alone markedly enhances cell proliferation and lnc-GAN1 overexpression alone remarkably decreases cell proliferation in A549 cells compared with blank or respective control treatments, whereas when both are overexpressed, the cell proliferation is not changed; downregulation of miR-26a-5p alone or lnc-GAN1 alone or both combination produces opposite effects on cell proliferation in H1299 cells. (**c**) miR-26a-5p overexpression alone significantly promotes cell cycle progression and lnc-GAN1 overexpression alone has the reverse effect on the cell cycle in H460 cells, whereas when both are overexpressed, the cell cycle is not changed; downregulation of miR-26a-5p alone or lnc-GAN1 alone or both combination produces opposite effects on cell cycle in H1299 cells. (d-e) In TCGA database, PTEN mRNA expression is significantly lower in lung squamous cell carcinoma than in normal tissue. (f) Protein-protein interaction network analysis of the differentially expressed genes induced by lnc-GAN1 overexpression indicates that PTEN protein plays a key role in the cell cycle protein interaction network. (**g**) PTEN can directly bind to Cyclin D1 and CDK4 proteins in H1650 cells, as detected by co-immunoprecipitation with antibody against PTEN and western blot.
